# Tea Consumption and Diabetes: A Comprehensive Pharmacological Review of Black, White, Green, Oolong, and Pu-erh Teas

**DOI:** 10.3390/plants14131898

**Published:** 2025-06-20

**Authors:** Ochuko L. Erukainure, Chika I. Chukwuma, Jennifer Nambooze, Satyajit Tripathy, Veronica F. Salau, Kolawole Olofinsan, Akingbolabo D. Ogunlakin, Osaretin A. T. Ebuehi, Jeremiah O. Unuofin

**Affiliations:** 1 Laser Research Centre, Faculty of Health Sciences, University of Johannesburg, Doornfontein 2028, South Africa; 2 Centre for Quality of Health and Living (CQHL), Faculty of Health and Environmental Sciences, Central University of Technology, Bloemfontein 9301, South Africa; 3 Department of Chemistry, Faculty of Natural and Agriculture Science, University of the Free State, Bloemfontein 9300, South Africa; n.jennifer33@gmail.com; 4 Amity Institute of Health Allied Sciences, Amity University, Noida 201301, Uttar Pradesh, India; 5 Department of Pharmacology, School of Medicine, Faculty of Health Sciences, University of the Free State, Bloemfontein 9300, South Africa; veronica.salau@yahoo.com (V.F.S.); kollyck@gmail.com (K.O.); 6 Phytomedicine, Molecular Toxicology, and Computational Biochemistry Research Laboratory (PMTCB-RL), Department of Biochemistry, Bowen University, Iwo 232101, Nigeria; akingbolabo.ogunlakin@bowen.edu.ng; 7 Department of Biochemistry, College of Medicine, University of Lagos, Lagos 101017, Nigeria; ebuehi@yahoo.com; 8 Department of Life and Consumer Sciences, University of South Africa, Florida Campus, Johannesburg 1709, South Africa; unuofinjeremiah@gmail.com

**Keywords:** blood glucose, *Camellia sinensis*, diabetes, tea catechins

## Abstract

Diabetes is one of the major non-communicable diseases whose physiological complications are linked with a higher risk of mortality amongst the adult age group of people living globally. This review article documents updated pharmacological evidence and insights into the antidiabetic mechanisms of green, black, white, oolong, and pu-erh teas via reported experimental and clinical models toward encouraging their use as a complementary nutraceutical in managing the biochemical alterations found in the onset and progression of diabetes. Peer-reviewed articles published in “PubMed”, “Google Scholar”, and “ScienceDirect” from 2010 and beyond that reported the antidiabetic, antilipidemic, and digestive enzyme inhibitory effects of the selected tea types were identified. The keywords used for the literature search comprise the common or scientific names of the tea and their corresponding bioactivity. Although teas portrayed different antidiabetic pharmacological properties linked to their bioactive components, including polyphenols, polysaccharides, and amino acids, the type of phytochemical found in each tea type depends on their processing. Green tea’s strong carbohydrate digestive enzyme inhibitory effect was linked with Ellagitannins and catechins, whereas theaflavin, a main ingredient in black tea, increases insulin sensitivity via enhancing GLUT4 translocation. Theabrownin in pu-erh tea improves FBG and lipid metabolism, while chemical components in white tea attenuate prediabetes-mediated reproductive dysfunctions by improving testicular tissue antioxidant capabilities. Based on the body of findings presented in this article, it is evident that integrating tea intake into daily food consumption routines could offer a promising practical solution to support human health and well-being against diabetes disease.

## 1. Introduction

In the twenty-first century, diabetes, a risky and financially damaging ailment that is on the rise in both industrialized and developing countries, poses a serious threat to public health. Diabetes was the fifth most prevalent cause of mortality in the US in 2000 [1]. More than 200,000 Americans with diabetes die each year from complications related to their condition, with coronary heart disease (CHD) or another form of cardiovascular disease (CVD) accounting for the majority of these deaths [2]. When compared to non-diabetic adults under the age of 45, diabetes significantly raises a person’s risk of peripheral vascular, ophthalmic, and renal disease as well as other chronic illnesses.

End-stage renal disease (ESRD), difficult pregnancies, sexual dysfunction, new-onset blindness, stroke, and heart failure are among the risks that are exacerbated by diabetes. Obesity, dyslipidemia, and hypertension are prominent CVD risk factors in people with diabetes [3]. Diabetes is more prevalent in older adults and members of specific racial groups, including Hispanics, African Americans, American Indians, and Alaskan Natives [4,5]. The changing demographics of the population, which include an increase in the proportion of a number of ethnic minorities, are a contributing factor in the rising prevalence of diabetes.

Diagnosed diabetes is today far more frequent than it was 40 years ago, both in the United States and around the world.

**Figure 1:**
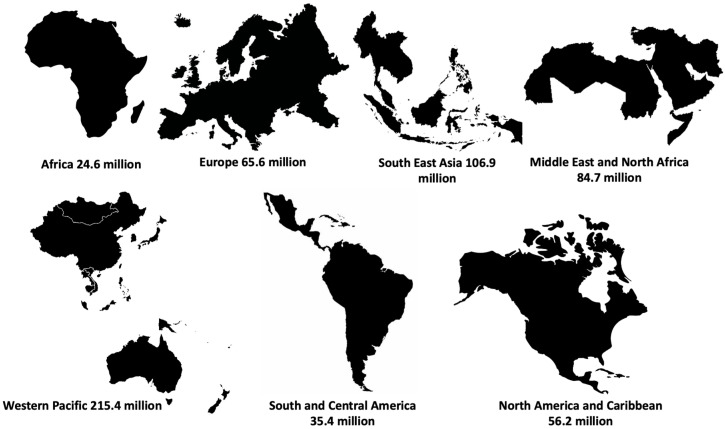
Number of adults with diabetes in IDF regions.

According to the International Diabetes Federation (IDF), there are 588.7 million adults living with diabetes, with the Western Pacific accounting for the highest number, and Africa the lowest (Figure 1) [6]. This number has been estimated to increase to 783 and 852.5 million by 2045 and 2050, respectively [6]. The IDF further accorded 3.4 million deaths in 2021 to diabetes [6]. Of all diabetes types, type 2 diabetes (T2D) accounts for over 90% of these cases.

This growth can be attributed to globalization which has had an impact on lifestyle changes in both developed and developing nations. Ironically, while medical progress has prolonged life by eliminating many infectious diseases, it has also led to an increase in diabetes prevalence globally [7].

### 1.1. Types of Diabetes

Type 1 diabetes (T1D) and type 2 diabetes (T2D) are the most common types of diabetes, with the latter being the most predominant. Other types include gestational diabetes mellitus (GDM), which occurs during pregnancy, and diabetes insipidus, which impairs the synthesis, transportation and/or release of vasopressin [8,9]. For this study, our focus is on T1D and T2D.

### 1.2. Type 1 Diabetes

Type 1 diabetes is characterized by insulin deficiency owing to the autoimmune-mediated selective destruction of the pancreatic β-cell, leading to hyperglycemia [10]. It is characterized by polydipsia, polyphagia, and polyuria, which are the most common form of its diagnosis [11,12].

The destruction of pancreatic β-cells by the body’s immune system is viewed as an inflammatory response due to the involvement of inflammatory cytokines, particularly those targeting β-cell autoantigens, macrophages, dendritic cells, B-lymphocytes, and T-lymphocytes [13,14]. Genetic predispositions also induce autoimmune destruction of the pancreatic β-cells, with the human leukocyte antigen (HLA) locus being the most predisposing [15]. MHC haplotypes and non-HLA loci can exacerbate the inflammatory and immune response to stimuli, thereby increasing the predisposition of β-cells to autoimmune destruction [16,17]. Environmental factors, including infant and adolescent diet types, vitamin D deficiency, viral infections, growth dynamics, exposure to toxins, ante- and perinatal risk factors, vaccinations, and stress, also significantly contribute to the development of T1D [16,17,18]. These environmental factors influence genetic predisposition, which triggers the activation of cytokines that lead to autoimmune destruction of pancreatic β-cells [19].

### 1.3. Type 2 Diabetes

Type 2 diabetes is a complex metabolic disorder with diverse characteristics, marked by a reduction in insulin secretion and/or its activity, resulting in elevated blood glucose levels (hyperglycemia) [20]. It arises from β-cell dysfunction, insulin resistance, and chronic inflammation of the pancreatic tissues.

Both genetic and environmental factors including physical activities have been linked to the development of T2D. Reduced physical activity and obesity cause a decrease in insulin sensitivity, which when combined with a genetic predisposition to insulin resistance prompts pancreatic β-cells to increase insulin production in an attempt to compensate for the ineffective insulin response [21]. Prolonged increases in insulin secretion will lead to a decrease in the functioning of pancreatic β-cells over time [21,22,23]. A rise in fasting plasma glucose levels then triggers insulin resistance, ultimately contributing to the development of diabetes [21].

**Figure 2:**
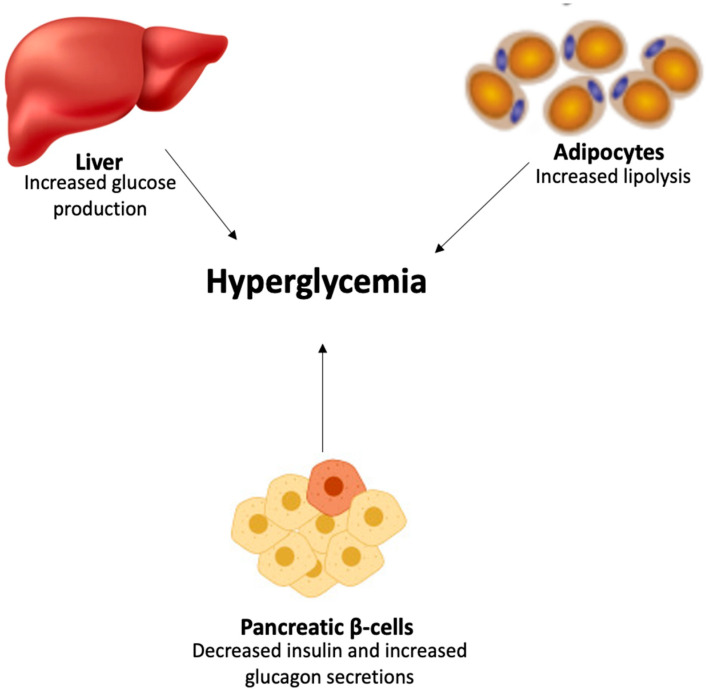
Onset of hyperglycemia in T2D.

Insulin resistance results in elevated hepatic glucagon levels and intensified hepatic glucagon responsiveness, prompting the liver to produce excessive glucose [21], thereby contributing to the onset of hyperglycemia in T2D (Figure 2). Insulin resistance also leads to a higher rate of lipolysis in the adipocytes, resulting in increased FFA plasma levels, which in turn exacerbate insulin resistance in the liver and muscle and contribute to the failure of pancreatic β-cells, ultimately causing hyperglycemia [21].

## 2. Management of Diabetes

Diabetes is often managed via pharmacological intervention combined with physical activities and modified diets and lifestyle. These interventions are aimed at reducing hyperglycemia and improving insulin secretion and sensitivity. Most pharmacological interventions are conventional commercial drugs and are associated with high costs and side effects [24,25]. These high costs and side effects have been recognized as a double-edged sword in the management of diabetes especially in developing countries with limited health facilitates and economic resources [26,27]. This has led to a continuous search for affordable treatments with little or no side effects, which has seen a paradigm shift towards medicinal plants and their products.

The use of medicinal plants in the treatment of diabetes and other diseases has been in practice from time immemorial. The efficacies of these medicinal plants have been attributed to their phytochemical constituents. Among these medicinal plants is *Camellia sinensis*, which is commonly known as tea.

At present, there is no thorough review known to us that has simultaneously documented the antidiabetic properties of various common tea types. This review was designed to document the pharmacological evidence from *in vitro*, *ex vivo*, *in vivo*, and clinical studies on the antidiabetic effects of green, black, white, oolong, and pu-erh teas. This could offer insights into the antidiabetic mechanism of tea and encourage its use as a complementary functional food and/or nutraceutical in managing elevated blood glucose levels.

## 3. Review Strategy

A literature search was carried out on “PubMed”, “Google Scholar”, and “ScienceDirect”, with the aim of identifying peer-reviewed published (mostly studies from 2010 and beyond) data reporting the antidiabetic, antilipidemic, and digestive enzyme inhibitory effects of the selected tea types. The keywords comprised a mixture of the name (either common or scientific) of the tea and the corresponding bioactivity. The search results were carefully chosen to align with the focus of this review. An analysis of the selected studies was conducted to identify the phytochemical properties and antidiabetic mechanisms of the different teas.

## 4. Camellia Sinensis

*Camellia sinensis*, often known as the tea plant, tea shrub, or tea tree, is a species of tiny, evergreen tree or shrub belonging to the family of flowering plants. Tea, a well-known beverage, is made from the plant’s leaves and leaf buds. It is a member of the *Camellia* genus, which has around 220 species that have been identified [28]. The tea plant is indigenous to East Asia, and it probably first appeared along the borders of north India, southwestern China, Myanmar, Cambodia, and north Burma [29]. However, it is now grown all over the world in tropical and subtropical areas such as Sri Lanka, Japan, Tanzania, and Kenya [30].

### Types of Teas

Black tea, white tea, yellow tea, green tea, oolong tea, and dark tea (which includes pu-erh tea) are among the several forms of tea [31].

White tea is one of numerous types of tea that typically uses young or barely processed *C. sinensis* plant leaves [32]. White tea has a flavor that is lighter than most green or conventional black tea since it is not rolled or oxidized. The name white tea refers to the white hairs that encircle the buds when they are picked [33]. White tea decoction has a pale yellow tint, similar to some green teas [33]. It has a mildly savory flavor with a tinge of sugar, as well as a fresh and green aroma [34]. The high concentrations of peptides, amino acids, and soluble sugar, which produce an average savory and sweetness flavor, and the decline in catechins and other polyphenols, which lessen the severity of astringency and bitterness, are responsible for these flavor qualities [34].

Yellow tea, also known as Chinese huángchá and Korean hwangcha, is an uncommon and pricey kind of tea [35]. The steps for manufacturing yellow tea are identical to those used to produce green tea, but they also include encasing, or sweltering, which gives the leaves a faint yellow hue as they dry [35]. Yellow tea is often categorized alongside green tea due to its relatively low level of oxidation. One of the main purposes of creating yellow tea is to eliminate the distinctively grassy aroma of green tea [35].

Green tea is made from freshly picked tea leaves and buds of *C. sinensis* that has been steamed or dried at a high temperature with caution to prevent the withering and oxidation of the polyphenolic compounds, which include flavanols [36]. The leaves of *C. sinensis* are initially steamed, then pan-fried, and subsequently dried to produce green tea. Because green tea is not fermented, it retains essential compounds known as polyphenols, which are believed to be the main reason behind many of its health benefits. Additionally, it has caffeine. Infusions manufactured from dried green tea leaves are recognizable by their vivid yellow or green color and tannic, bitter flavor [37]. Green tea contains greater quantities of l-theanine than previously thought because there is no fermentation involved in its production [38]. According to Zhang et al. [39], the bitterness is recognized as a significant characteristic that adds to the sensory distinction of green tea. Green tea is processed in a way that retains the catechin content of fresh tea leaves because bitterness is governed by catechin content, especially gallated catechins, and low catechin concentration may cause a degradation of the distinctive green tea flavor [39].

Oolong tea is a classic semi-oxidized Chinese tea prepared from the leaves and buds of *C. sinensis* [40]. The leaves are oxidized, curled, and twisted when the plant withers in sunlight. Most oolong teas, particularly those of good grade, use distinctive tea plant cultivars that are only used for specific types. Depending on the variety and production method, the degree of oxidation can range from 8 to 85%, which fluctuates according to the chosen amount of time before fire [40]. Oolong tea comes in a wide range of flavor profiles. Depending on the horticulture and method of production, they can have a variety of flavors, including sweet and fruity with honey scents, woody and heavy with roasted aromas, or green and fresh with complex flavors [40,41]. Oolong leaves naturally contain caffeine, although the quantity in tea varies according to the region where the tea is grown and the method of processing. Oolong tea frequently contains more caffeine than most green teas [41].

Dark tea, often referred to as post-fermented tea or fermented tea, is a type of tea that has gone through microbial fermentation for a period ranging from a few months to numerous years [39]. As a result of the process, the tea leaves also undergo exo-oxidation, which is microbially catalyzed, and endo-oxidation, which is obtained from the enzymes in the tea leaves themselves [39]. Oxidation results in a darkening of the tea leaves and the resulting beverage. Tea leaves’ chemistry is changed during fermentation, which has an impact on the organoleptic properties of the tea that is produced from them [42]. The process of fermentation alters the tea’s aroma and often softens its flavor by lowering astringency and bitterness and enhancing mouthfeel and aftertaste [42]. The microorganisms may also create compounds that are advantageous to health. It is also possible to create materials like ethyl carbamate (urethane). Pu-erh tea infusion has a reddish to brownish red color because of its high theabrownin concentration [39]. Pu-erh tea has a full-bodied, complex, and mellow flavor as well as a putrid taste and a velvety-smooth mouthfeel, according to Zhang et al. [39]. These flavor characteristics in pu-erh tea may be brought on by theabrownins and the modest quantities of natural polyphenols present [39].

Black tea is a variety of tea that has undergone more oxidation than green, oolong, white, or yellow teas [43]. Compared to other types of tea, black tea generally possesses a more robust taste [43]. Green tea leaves that were once fresh are converted into dried tea leaves that are dark brown in color [33]. A dark brownish-red infusion is produced when dried black tea leaves are brewed [44]. Black tea infusions’ characteristic color and flavor are mostly due to theaflavins and thearubigins [45]. According to Yao et al. [46], thearubigins provide a rich mouthfeel and a brown-red color, whilst theaflavins add to the tannic and brisk flavor and the golden color.

## 5. Phytochemical Properties of Teas

**Figure 3:**
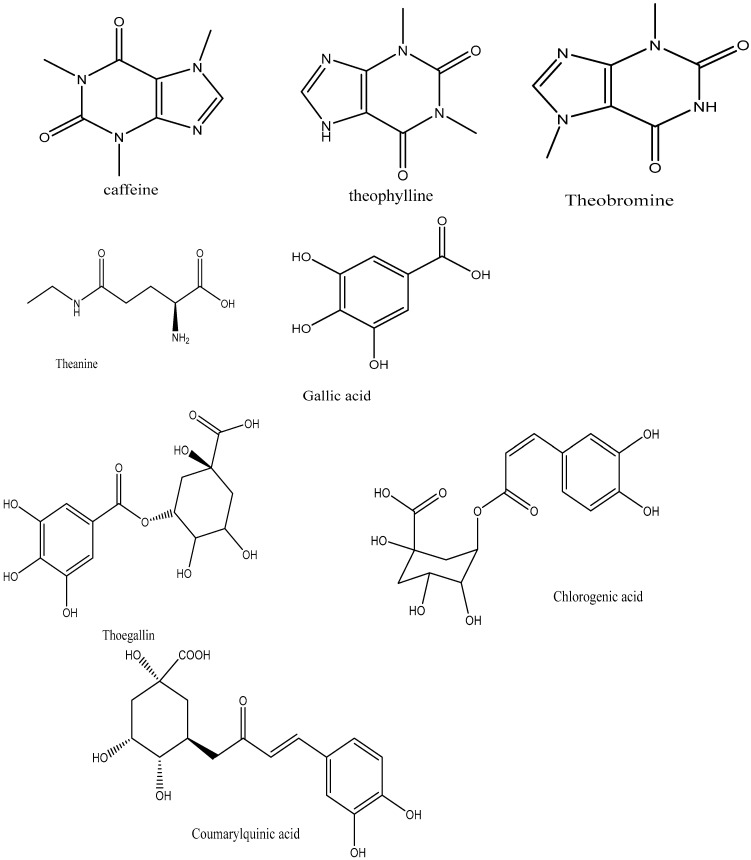
Structure of tea methylxanthines and gallic acid and the depsides.

Dried tea leaves are manufactured from fresh tea leaves obtained from the *C. sinensis* plant, which is responsible for producing various types of tea. The main ingredients in fresh tea leaves are proteins and carbohydrates like cellulose [47]. High concentrations of these enzymes are crucial for the digestion of black tea since they are accountable for catechin oxidation and are included in the protein component [48]. Another important element is l-theanine, the primary amino acid found in tea leaves and a N-methylated derivative of glutamine [33]. Fresh tea leaves contain caffeine and other methylxanthines, along with trace amounts of theobromine and theophylline (Figure 3) [49].

**Figure 4:**
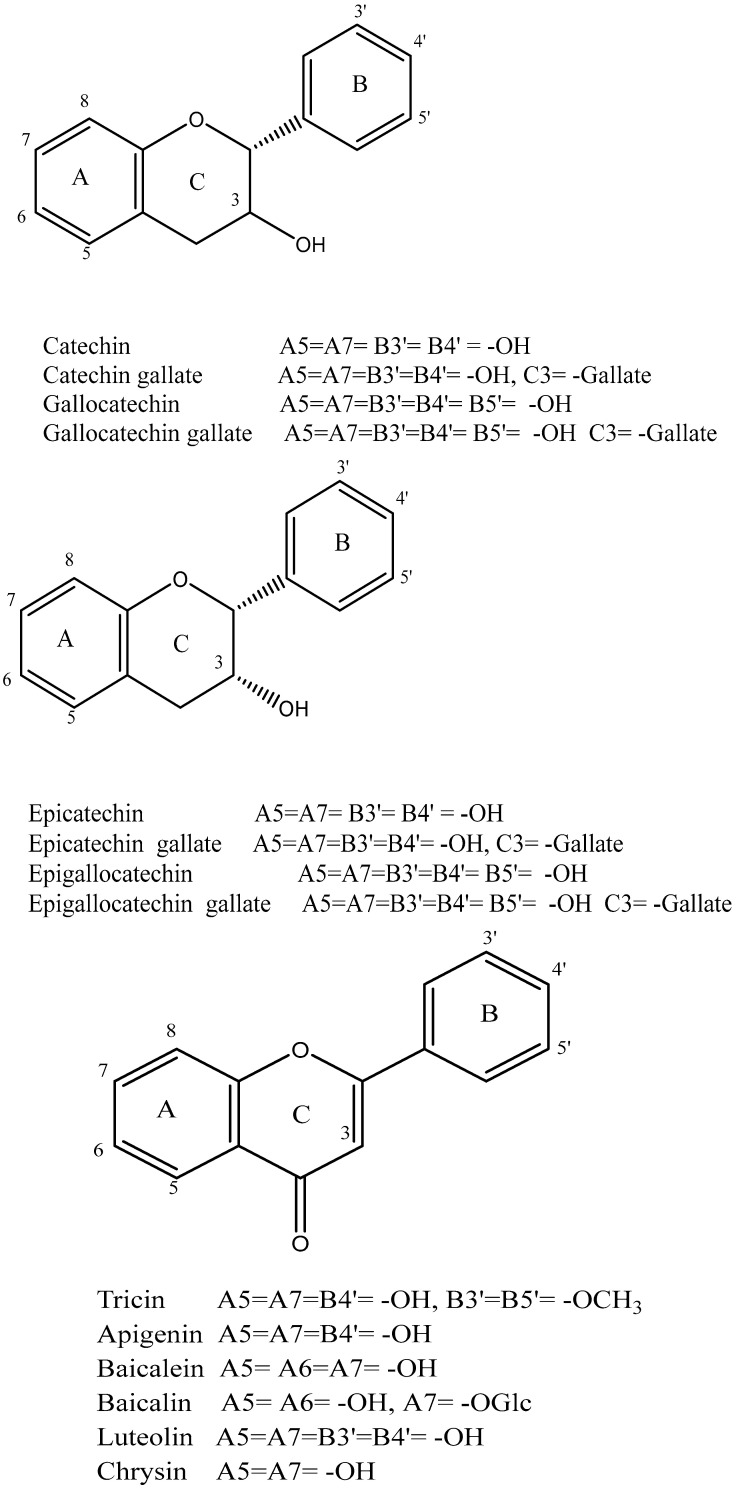

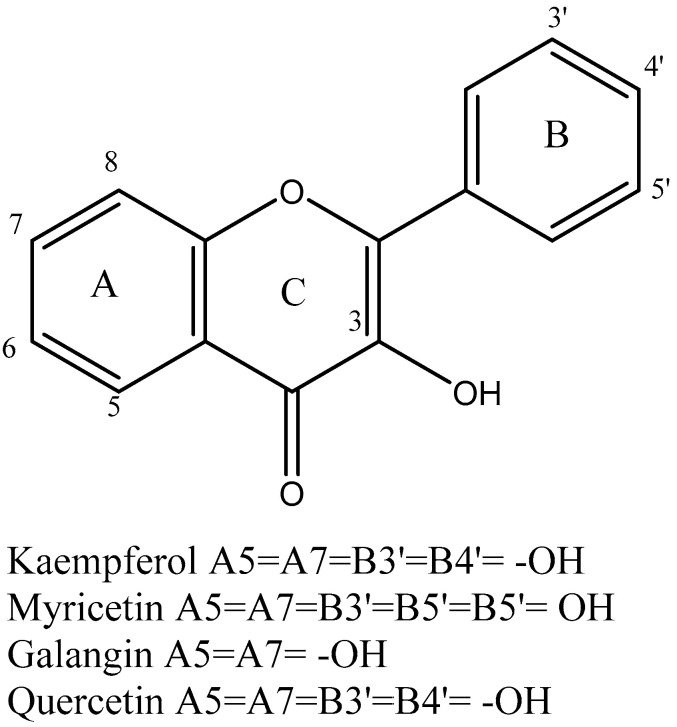
Structure of flavanols, flavonoids, and flavones identified in tea.

Polyphenol is the other essential component found in tea leaves. In addition to catechins (flavan-3-ols) and proanthocyanidins, which are catechin dimers and oligomers, the principal class of polyphenols found in fresh tea leaves is known as flavanols [50]. Other polyphenol families, including flavonols and flavones, are also present, albeit in much lesser quantities [33]. The flavonols kaempferol, quercetin, and myricetin, (Figure 4) as well as their glycosides, are present in fresh tea leaves. Apigenin is a flavone.

Graham [48] asserted that phenolic substances including gallic acid and depsides are also present in freshly brewed tea leaves (Figure 1). The depsides present in tea leaves are formed by the condensation of theogallin, a quinic acid ester of gallic acid, and chlorogenic acid, two hydroxy acids [51]. Black tea and other fermented teas must be processed using gallic acid [48].

Catechins are the main polyphenols found in fresh tea. The major catechins are the four (–)-stereoisomeric forms, (−)-epicatechin (EC), (−)-epigallocatechin (EGC), epicatechin gallate (ECG), and (−)-epigallocatechin gallate (EGCG). Fresh tea leaves also contain the (+)-stereoisomeric forms, such as (+)-gallocatechin (GC) and (+)-catechin (C), albeit in much lower amounts (Figure 4) [52]. Several studies have reported EGCG as the most prevalent catechin in fresh tea leaves [53]. These studies ranked the quantities of the various catechins as EGCG > EGC > ECG > EC > C. The levels of catechins can vary based on factors such as harvesting season, leaf age, harvesting technique, tea plant type, and cultivar [52].

### 5.1. White Tea

The withering procedure is the main cause of the chemical alterations in white tea. Cell membranes increasingly deteriorate during the withering process, permitting catechin to form relationships with peroxidase and polyphenol oxidase (PPO) [34]. In light of this, it is anticipated that catechin may undergo oxidation and produce dimeric molecules following withering [34]. The quantity of free amino acids like phenolic acids, theanine, and caffeine may also change during the withering process [34]. Due to the use of heat during drying, which can result in the degradation of components like catechins, polyphenols may also be changed. However, the effect of drying on phytochemicals is anticipated to be much less pronounced than that of withering [34].

### 5.2. Green Tea

The content of catechins in green tea is expected to change minimally throughout processing since oxidative enzymes are turned off to prevent catechin oxidation and no fermentation occurs [52]. Catechins can make up as much as thirty percent of the dry weight of green tea, similar to the composition of newly picked tea leaves [51]. Green teas that have wilted may retain lower catechin levels despite the presence of active PPO, which in turn allows for catechin oxidation to occur [54]. Donlao and Ogawa [55] reported that both pan-firing and drying at elevated temperatures can lead to a reduction in total catechin content. Eight catechins have been found to be present in green teas as contrasted to fresh tea leaves (Figure 4), with EGCG being the most prevalent catechin [53]. Compared to fresh tea leaves, green tea has higher levels of (−)-gallocatechin gallate (GCG) and (−)-catechin gallate (CG) [56]. Epimerization of EGCG and ECG, typically resulting from pan-frying and drying at high temperatures, is primarily responsible for this occurrence [53,55].

### 5.3. Oolong Tea

**Figure 5:**
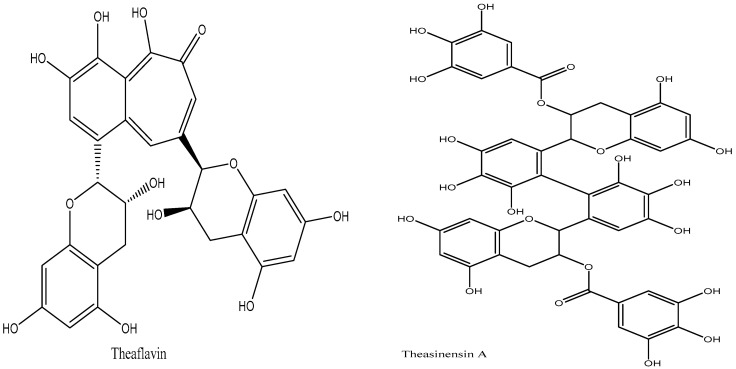
Structure of theasinensins A and theaflavins.

The catechins in oolong tea should oxidize into theasinensins and theaflavins (Figure 5), which are colorless catechin dimers [57,58]. Although semi-fermented teas have significantly less catechin oxidation than completely fermented teas, black tea is a fully fermented tea [59]. Theasinensins are more commonly formed in oolong teas that have undergone light fermentation processes, in contrast to theaflavins, which are produced in teas that have undergone more extensive fermentation procedures [60]. Additionally, it is anticipated that the catechins undergo oxidation into oolongtheanins, which are distinctive dimers of oolong teas [61]. According to Chen et al. [62], processing is also accountable for the creation of volatile chemicals that give oolong tea its distinct aroma. These modifications include the conversion of lipids, fatty acids, and carotenoids to aromatic compounds as well as additional hydrolysis and oxidation reactions.

### 5.4. Black Tea

The primary chemical modification that occurs during the preparation of black tea is the oxidation of catechins to create thearubigins and theaflavin. Upon cell destruction, previously separated catechin in the cytoplasm, chloroplast PPO, and cell wall peroxidase come into contact, resulting in catechin oxidation and theaflavin formation [63]. Catechins undergo significant oxidation during fermentation, which leads to the formation of theaflavins [64]. The primary theaflavins present in black tea are theaflavin, theaflavin 3-gallate, theaflavin 3′-gallate, and theaflavin 3,3′-gallate [65]. Theaflavins are the oxidized dimers of catechin monomers.

The high-intensity maceration process results in more pronounced and accelerated catechin oxidation and theaflavin production in black CTC teas. The damage to rolled tea leaves during processing likely results in lower theaflavin production in black orthodox teas [66]. Catechins can oxidize to form theasinensins instead of just theaflavins. Theasinensins A and D, which are isomeric dimers of EGCG, are the main theasinensins present in black tea. The majority of theaflavins are produced when roughly 75% of the catechins in tea leaves undergo complete fermentation, yet the quantity of theaflavins in black tea is not substantial. This is most likely caused by theaflavin oxidation, which produces thearubigin [52]. Black tea contains a class of polyphenols called thearubigins, which is still not well understood. According to Harbowy et al. [51], they are reddish-brown pigments that are unable to be separated by high-performance liquid chromatography (HPLC) and are produced by theaflavin oxidation and polymerization, which is aided by peroxidase [67].

The production of gallic acid—which can be biosynthesized in tea leaves through a variety of processes including oxidation of the sidechain of 3,4,5-trihydroxycinnamic acid, dehydrogenation of shikimic acid with 3-dehydroshikimic acid as an intermediate, and hydroxylation of protocatechuic acid—is another significant chemical alteration that occurs during the manufacturing process of black tea [68]. Another source is fermentation, which results from tannase, an inducible enzyme released by microorganisms, breaking down tannic acid [59].

### 5.5. Dark Tea (Pu-erh Tea)

The defining characteristic of dark ripened tea is the post-fermentation process, a bacterial fermentation method. The production of extracellular enzymes by the microorganisms, which catalyze the degradation, oxidation, and condensation of various tea ingredients, results in a variety of chemical changes during this process [69]. According to several studies, microbial fermentation is predicted to have an impact on phytochemicals such caffeine, gallic acid, amino acids like theanine, and catechins [70,71].

**Figure 6:**
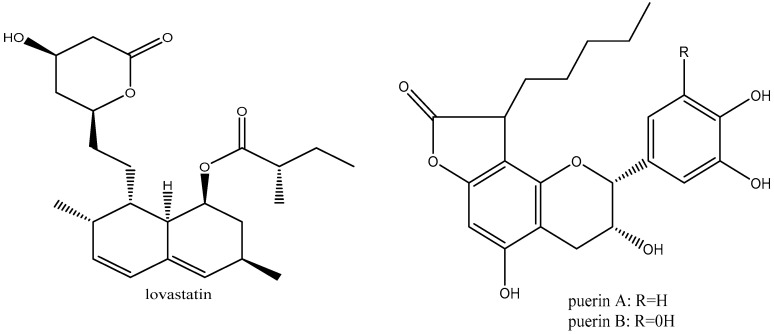
Structure of lovastatin and puerins.

The key bioactive compound found in pu-erh tea is theabrownin, a dark brown coloring that appears in dark tea varieties [72]. Theabrownins also have unidentified chemical structures, although spectroscopic study has shown that they are complicated molecules made up of lipids, phenols, proteins, flavonoids, and carbohydrates [73]. According to research by Zhang et al. [74], theabrownins are produced as a result of catechin degradation and oxidation. Finally, some new compounds have been identified in dark tea. These compounds comprise of effective HMG-CoA reductase inhibitors like puerins and lovastatin (Figure 6), which both significantly lower the amount of LDL cholesterol in the body [74].

## 6. The Diabetes-Remedying Effect of Teas

### 6.1. White Tea

The antidiabetic properties of white tea are summarized in Table 1.

#### 6.1.1. *In Vitro* Studies

Tenore et al. assessed how white tea polyphenols affected lipids and the metabolism of glucose in HepG2 cell lines [75]. The polyphenols significantly showed reduced activities in the absorption of both glucose and lipid, while increasing HDL content and LDL receptor binding activity. They also suppressed lipase activity with a concomitant reduction in triglyceride levels, thus demonstrating the health advantages of white tea in metabolic syndrome.

The antioxidant and antidiabetic activity of the methanolic extract of white tea has been investigated by Kalauni and Sharma [76]. A DPPH assay was used to measure antioxidant properties, and the results showed that the methanolic extract of white tea had powerful antioxidant properties comparable to conventional ascorbic acid. The extract’s antidiabetic ability was further demonstrated by inhibition of α-amylase activities.

Esposito et al. examined the total phenolic (TPC), catechin (EGCG, ECG, EGC, and EC) content, as well as α-glucosidase inhibitory actions of preparations of three distinct tea varieties (green, white, and oolong) [77]. When compared to oolong and green tea, white tea had much greater TPC, which suggests that its fresh, young leaves have a higher concentration of EGCG and ECG catechins. This investigation revealed a robust correlation between the amount of these catechins in white tea infusion and a significant inhibitor of α-glucosidase. This inhibition was also shown to be more potent than the FDA-approved medication acarbose.

#### 6.1.2. *In Vivo* Studies

In streptozotocin (STZ)-induced diabetes, Isalm investigated the effects of a 0.5% aqueous extract of white tea [78]. Diabetic male Sprague–Dawley rats were treated with white tea and compared to a normal control and diabetes control. In comparison to the controls, white tea significantly improved blood glucose level and tolerance, while concomitantly reducing serum levels of cholesterol and LDL cholesterol and boosting body weight, liver weight, and glucose, serum insulin, and fructosamine levels.

Nunes et al. examined the impact of daily white tea drinking on the brains of prediabetic rats [79]. The expression levels of GLUT, phosphofructokinase-1, lactate dehydrogenase (LDH), and monocarboxylate transporter 4 have been assessed along with the brain cortex’s metabolism profile. Regular consumption of white tea improved insulin sensitivity and glucose tolerance. White tea also modulated the cortex’s glycolytic profile by improving GLUT4 expression and lactate and alanine levels. White tea also increased the cortex’s antioxidants activities, restored protein level and catalase expression, and mitigated lipid peroxidation. Furthermore, daily consumption of white tea enhanced the metabolic and oxidative profile of prediabetic the rats’ cerebral cortex, indicating that it may be an effective, secure, and affordable method for avoiding diabetic-mediated complications in the brain cortex.

According to Alves et al.’s hypothesis, white tea consumption by prediabetic rats enhanced their hearts’ glycolytic and oxidative profiles [80]. Wistar male prenatal rats were separated into control and prediabetes (PrDM) groups, with the latter receiving an injection of STZ. After one month, the PrDM rats were split in half, with each group consuming water or white tea for two months. Consumption of white tea enhanced myocardial acetate and alanine contents, protein oxidation levels, and glucose tolerance and insulin sensitivity in PrDM rats. It also improved the rats’ overall metabolic conditions, while mitigating most of the studied toxic effects on the heart.

Dias et al. postulated that consuming white tea by prediabetic mice could lessen the metabolic changes in their testicular and epididymal tissues, maintaining sperm purity [81]. White tea infusion was administered to STZ-induced prediabetic rats. After intervention period, white tea reversed prediabetes-mediated GLUT 3 protein expression and decreased LDH and lactate concentration in testicular tissues. The tea also improved testicular alanine levels. Additionally, white tea restored sperm viability and increased epididymal sperm mobility.

Al-Shiekh et al. investigated how white tea extract affected the function of antioxidant enzymes [82]. Comparing diabetic rats to non-diabetic control rats, white tea extract showed a significantly higher level of serum and liver SOD, GSH-px, and catalase activity, thus giving credence to the antioxidant properties of white teas in diabetes.

White tea ingestion by prediabetic rats enhanced the heart glycolytic and oxidative description, according to Xia’s hypothesis [83]. Wistar male prenatal rats were separated into control and prediabetes (PrDM) groups, with the latter receiving a shot of STZ. After one month, the PrDM rats were split in half, with each group consuming water or WTEA for a period of two months. The phytochemical composition of WTEA was discovered. After that, tests for resistance to insulin and tolerance to glucose were performed on rats. Heart oxidative and glycolytic patterns were identified. Diabetes prediabetes reduced lactate and acetate levels, lactate dehydrogenase activity, and mRNA expression of glucose transporters (GLUT1 and GLUT3) in heart tissue. Diabetes also reduced the heart’s capacity for antioxidants, which raised protein and lipid oxidation and peroxidation, respectively. WTEA ingestion enhanced myocardial acetate and alanine contents, protein oxidation levels, and glucose tolerance and insulin sensitivity in prediabetic rats. WTEA consumption improved the prediabetic rats’ overall metabolic condition and mitigated the majority of the harmful effects on the heart that were assessed.

Oliveira et al. investigated if white tea ingestion may attenuate testicular oxidative stress and maintain sperm quality in prediabetic mice [84]. For that reason, 30-day-old STZ-induced prediabetic rats received white tea for two months. In rats with prediabetes, white tea intake increased insulin sensitivity and glucose tolerance. While glutathione concentration and redox status were unaffected, the tea boosted the testicular antioxidant activities and mitigated protein oxidation and lipid peroxidation. White tea increased sperm quantity and enhanced its quality (motility, survival, and abnormalities).

Amanzadeh et al. examined the impact of aqueous white tea extract on hyperglycemia and lipidemia in STZ-induced diabetic rats [85]. White tea improved blood glucose level and modulated lipidemia by reducing serum levels of LDL, triglycerides, and cholesterol, and increasing HDL level. The improved blood glucose level corresponds to a previous study on the antihyperglycemic properties of white tea in diabetic rats [86].

Silveira et al. investigated at how prediabetes affected the lungs of male Wistar rats and if regular consumption of white tea could improve the tissue’s antioxidant profile [87]. According to this data, PrDM elevated protein nitrification and lipid peroxidation while decreasing lung SOD, GPx, and histone H2A concentration. Regular consumption of white tea improved the lung’s antioxidant enzymes activities and restored histone H2A levels, while mitigating lipid peroxidation and protein nitrification.

#### 6.1.3. Clinical Trials

In a controlled experiment, Yaghobian et al. examined the impact of white tea intake on individuals with type 2 diabetes mellitus, as well as whether they were also performing aerobic exercise or not [88]. Forty-nine T2D patients were randomly divided into four groups: control, white tea, aerobic exercise, and aerobic exercise with white tea. Six months were spent on the treatments. At the start and conclusion of the study duration, various physiological parameters, including body weight, body mass index (BMI), fat mass, maximum oxygen intake (VO_2_Max), and blood pressure levels, were recorded. On the same days, blood samples were collected through venipuncture to measure triglycerides, cholesterol levels, LDL, HDL, and blood glucose level. White tea improved blood pressure, VO_2_Max, and BMI. In comparison to the start of the experiment, consumption of white tea led to decreased levels of glucose, insulin, LDL, cholesterol, and triglycerides, and substantially increased HDL levels. Thus, regular intake of white tea when combined with aerobic exercise can have a positive overall impact on the blood pressure, VO_2_Max, lipid profile, and glycemic indices in women with T2D.

**Table 1:** Antidiabetic properties of white tea.

Cell Lines/Tissue/Animal Model/Human	Study	Treatment	Antidiabetic Mechanism	Reference
HepG2 liver carcinoma cells	*In vitro*	Cells seeded culture plates were incubated in assay solution with 0.5 mL of white tea infusion	Tea infusion suppressed cholesterol and glucose uptake in cells Decreased cells’ lipase enzyme activity Elevated cells’ LDL receptor binding activity Elevated the concentration of HDL released by cells into culture medium	[75]
Enzyme inhibition and free radical scavenging		10–100 µg/mL of white tea methanol extract was incubated with DPPH or α-amylase carbohydrate digestive enzyme solution	The tea extracts significantly scavenged DPPH radicals and inhibited α-amylase enzyme	[76]
		0.016–0.25 mg/mL of white tea infusion was incubated with α-glucosidase enzyme at 37 °C	The tea dose-dependently inhibited α-glucosidase enzyme	[77]
		0.2 mL or 0.01 mL of white tea cold and hot infusions were incubated with ABTS cation or serum LDL (100 mg protein/mL) in 96-well plates	The infusions significantly donated electrons to ATBS and lowered LDL production of conjugated diene	[89]
Sprague–Dawley rat blood DPP-IV models	*Ex vivo*	Blood sample was treated with 5–500 µg/mL white tea n-hexane, ethyl acetate, and methanol fractions	The fractions inhibit blood DPP-IV enzyme activity.	[90]
STZ-induced diabetes in male Sprague–Dawley rats	*In vivo*	Animals were given daily fresh solution of 0.5% white tea aqueous extract for 4 weeks	Treatment with the plant extract significantly increased liver weight and glycogen content Reduced cholesterol and LDL concertation in the serum Improved the overall glucose tolerance in the treated diabetic animals	[79]
STZ-induced diabetes in male Wistar rats		Rats were given white tea filtered infusion (1 g/100 mL) *ad libitum* for 2 months	The infusion improved cardiac tissue antioxidant profile Improved GLUT-1 and GLUT-3 gene expression in heart tissue Enhanced lactate dehydrogenase activity in cardiac tissue Elevated cardiac alanine and acetate levels in the rats	[80]
			The white tea product increased testes lactate dehydrogenase activity Improved sperm viability and motility Restored phosphofructokinase 1 activities in testicular tissue to a near-normal level	[81]
			White tea infusion improved the overall antioxidant capacity in the brain tissue. Decreased GLUT-1 and GLUT-3 protein expression levels in the brain cortex Reduce alanine concentration in the brain of diabetic animals	[79]
		Animals were gavaged daily with 5.1% white tea aqueous extract for 30 days	The plant extract significantly lowered glucose, cholesterol, TAG, and LDL but increased HDL levels in rat serum	[85]
STZ-induced diabetes in albino rats		Rats were administered daily with 2% *w*/*v* white tea aqueous extract in drinking bottles for 4 weeks	The tea significantly increased glutathione peroxidase, SOD, and catalase enzyme activities in liver and serum samples of diabetic animals	[82]
STZ-induced prediabetes in male Wistar rats		Rats were allowed to drink filtered white tea infusion (1 g/100 mL) *ad libitum* for about 2 months	Treatment with the tea considerably lowered serum glucose level Reduced protein carbonyl and lipid peroxidation concentrations in testicular tissue Significantly increased motility and concentration of sperm cells from the epididymis	[84]
			Tea extracts elevated lung tissue antioxidant enzymes’ activity Increased histone H2A protein expression in the lung Suppressed protein nitration in lung tissue	[87]
STZ–nicotinamide-induced diabetes in Sprague–Dawley rats		Rats were orally administered doses of 50, 100, and 200 mg/kg BW white tea ethanol extract for 14 days	Tea extracts lowered serum fasting blood glucose in experimental animals	[86]
Individuals with type 2 diabetes	Clinical trials	Females combined daily consumption of 150 mL of white tea infusion (made from 1 tea bag) with aerobic training for 6 months	Treatment with the tea infusion in combination with aerobic training remarkably improved serum lipid profile Decreased body fat, blood pressure, and fasting blood glucose	[88]

### 6.2. Green Tea

The antidiabetic properties of green tea are summarized in Table 2.

#### 6.2.1. *In Vitro* and *In Vivo* Studies

Tolmie et al. investigated the presence of ellagitannins in commercial green and purple teas, as well as the potential antidiabetic effects of these compounds and their metabolites, urolithins [91]. Corilagin, strictinin, and tellimagrandin I, three ellagitannins found in commercial teas, were quantified using targeted UPLC-MS/MS. Ellagitannins, which include corilagin, strictinin, and tellimagrandin I, were found to be potent inhibitors of α-amylase and α-glucosidase with Ki values that were much lower than acarbose. Commercial green-purple teas with very high corilagin contents were discovered to be ellagitannin sources. These ellagitannin-containing commercial purple teas were shown to be potent α-glucosidase inhibitors. The ability of urolithin A and urolithin B to boost glucose absorption in adipocytes, muscle cells, and hepatocytes was comparable to that of metformin. Additionally, urolithin A and B decreased lipid accumulation in adipocytes and hepatocytes compared to metformin. According to the research, the studied commercial teas have antidiabetic properties and are readily accessible and inexpensive.

Using an *in vitro* digestion model with Caco-2 cells, Chung et al. investigated the synergistic impact of green tea extract (GTE) and crude green tea polysaccharides (CTP) in suppressing glucose transfer following consumption of rice starch [92]. In comparison to the control, co-digestion of rice starch with GTE, CTP, or GTE + CTP digested less starch into glucose. After 120 min of incubation, GTE + CTP substantially suppressed glucose transfer from digesta to Caco-2 cells. GTE, CTP, or GTE + CTP had no effect on the gene expression of intestinal glucose transporters, except for the elevation in GLUT2 caused by GTE. The results showed that GTE + CTP decreased the digestion of rice starch and delayed intestinal absorption of glucose. Thus, demonstrating the potential of green tea polysaccharides as a postprandial hypoglycemia agent.

Kobayashi et al. [93] investigated the interference of green tea polyphenols with SGLT1 in intestinal epithelial cells through a competitive mechanism. Green tea polyphenols significantly reduced the transportation activity of SGLT1, with the galloyl groups in polyphenols such as epicatechin gallate (ECg) and epigallocatechin gallate (EGCg) having the strongest inhibitory properties. ECg suppressed SGLT1 competitively, even though ECg itself was not transferred by SGLT1 in brush border membrane vesicles from the small intestinal tract of rabbits. The current findings imply that tea polyphenols like ECg interact with SGLT1 as antagonist-like molecules, which potentially regulate the intestinal tract’s absorption of dietary sugar.

The effects of green tea ethanol extract (GTE) and polysaccharide fragments from green tea (PFGs) on the breakdown of wheat starch, microstructural alterations, and intestinal transit of glucose were investigated by Oh et al. [94]. Water-soluble polysaccharide (WSP), water-soluble polysaccharide–pectinase (WSP-P), and water-insoluble polysaccharide–alkali soluble (WISP-Alk-Soluble) considerably reduced the quantity of resistance starches (RSs). Intestinal glucose from digested wheat starch was transferred 2.12–3.50 times less than in the individuals in the control group. According to this study’s findings, meals containing starch may incorporate water- and alkali-soluble PFGs as prospective additives to reduce starch hydrolysis and regulate postprandial blood glucose levels.

In order to manage postprandial blood glucose levels, Xu et al. extensively evaluated the role of the key components of GTE in modulating glucose in the intestinal transit and the digestion of carbohydrates [95]. According to each catechin concentration in the GTE detected with high-performance liquid chromatography, a catechin mixture (CM) of seven catechins and epigallocatechin gallate (EGCG), was developed. In a cell-free system, the inhibitory potency of GTE, CM, and EGCG on α-amylase or α-glucosidase was compared. Catechins were primarily responsible for the inhibitory potency of GTE, with EGCG accounting for at least 80% of GTE’s α-amylase inhibitory activity and 90% of its α-α-glucosidase inhibitory activity. Additionally, EGCG quenched the fluorescence of the digestive enzymes, revealing that EGCG–amylase had a binding site of 1.2 and EGCG–glucosidase had a binding site of 2.0. In a Caco-2 monolayer system, the inhibiting efficacy of GTE, CM, and EGCG on glucose uptake was evaluated. CM exhibited higher inhibition than EGCG, whereas there was no discernible difference between CM and the GTE, thus demonstrating that green tea catechins are responsible for the tea’s putative postprandial hypoglycemic effects.

Chen et al. studied the effects of chronic administration of green tea (GT) on the body’s composition, glucose tolerance, and the expression of genes involved in energy metabolism and the balance of lipids in comparison to black tea (BT) and isolated EGCG [96]. Administration of GT led to decreased body fat and increased glucose tolerance while enhancing the expression of genes involved in fatty acid synthesis (SREBP-1c, FAS, MCD, and ACC) and oxidation (PPAR-, CPT-1, and ACO) was enhanced in the liver. It also suppressed Pref-1, C/EBP-α, and PPAR-α levels in were in perirenal fat, with no effect on the liver’s triacylglycerol level. Thus, demonstrating the potential of GT in improving glucose tolerance and reducing body weight.

Using a model of severe T1D, Ladeira et al. investigated if GT infusion has favorable effects on the kidney, irrespective of glycemic management [97]. Daily administration of GT (100 mg/kg BW) to STZ-induced diabetic young rats for 42 days led to improved glucose and glycogen metabolism while attenuating hypoxia and apoptosis. It also improved pathological characteristics by retaining glomerulus morphology and reducing in kidney capacity degradation.

In diabetic rats consuming a high-fat diet containing streptozotocin, Sundaram et al. investigated the antidiabetic effects of green tea extract (GTE) on key carbohydrate-metabolizing enzymes [98]. Daily oral administration of GTE (300 mg/kg BW) to diabetic rats for a period of 30 days caused a substantial decrease in blood glucose and HbA1c levels, as well as elevation in insulin and hemoglobin levels. GTE also improved glucose metabolism by increasing the activities of hexokinase, fructose-1,6-bisphosphatase, glycogen phosphorylase, pyruvate kinase, lactate dehydrogenase, glucose-6-phosphatase, dehydrogenase, and glycogen synthase in the liver of diabetic rats. Furthermore, the tea extract increased glycogen levels in the muscles and liver, indicating a reduction in blood glucose level.

Otton et al. analyzed whether alterations in the miRNA profile in white adipose tissue are the cause of green tea’s (GT) weight-loss benefits [99]. Treatment with GT (500 mg/kg BW/12 weeks) reduced weight growth, diminished adiposity, suppressed inflammation, and enhanced insulin sensitivity in mice fed a high-fat diet for sixteen weeks.

Using adiponectin knockout mice as a model, Bolin et al. investigated the involvement of adiponectin in the thermogenic effects of GTE [100]. Treatment of 3-month-old male C57Bl/6 knockout (AdipoKO) and wild-type (WT) mice with GT extract reduced body weight, adiposity index, adipocyte size, and insulin resistance while reversing consequences of obesity in WT mice; however, the extract was ineffective in reverting these indicators in the AdipoKO mice.

Ortsäter et al. investigated whether dietary supplement intake with GTE epigallocatechin gallate (EGCG) hinders the development of an intolerance to glucose in db/db mice [101]. For a period of ten weeks, young (7-week-old) db/db mice were randomly assigned to meals enriched with or without EGCG or rosiglitazone. EGCG enhanced glucose tolerance and boosts insulin release in response to glucose. Supplementing with EGCG decreased the proportion of pathologically altered islets of Langerhans while increasing the quantity and size of islets and expanding the pancreatic endocrine region. These were accompanied by decreased oxidative stress in the islet endoplasmic reticulum, thus indicating that EGCG from GT protects islet structure and improves glucose tolerance in T2D.

Holidah et al. investigated the impact of GTE on blood glucose level and the liver histopathology in mice with diabetes [102]. In male Balb/C adult mice (20–30 g; 2–3 months old), GTE caused a reduction in blood glucose levels while maintaining the ultrastructural integrity of the liver.

In renal and hepatic tissues of diabetic rats, Thomson et al. investigated how GT might affect oxidative damage and the levels of the angiotensin II AT1 receptor [103]. In STZ-induced diabetic rats, treatment with green tea significantly attenuated renal and hepatic oxidative stress by elevating total antioxidant and catalase levels and suppressing malondialdehyde levels and AT1 receptor labeling. Abolfathi et al. also reported a similar effect where GT enhanced SOD, catalase, and GSH-Px activities in hepatic tissues of STZ-induced diabetic rats [104]. These studies demonstrated the ability of GT to suppress the damaging consequences of excessive angiotensin II signaling and oxidative stress in diabetes-mediated hepatic and renal tissues.

Hininger-Favier et al. evaluated the effect of GTE on antioxidant markers and insulin sensitivity in mice with insulin resistance [105]. For a period of six weeks, 10 Wistar rats were given either a high-fructose diet (FD) or the same diet (FD) plus 1 or 2 g of GT solids/kg. Administration of additional tea solids led to reductions in glycemia, insulinemia, and triglyceridemia while concomitantly suppressing oxidative stress by attenuating DNA oxidative damage, sulfhydryl (SH) group oxidation, and plasma lipid peroxidation.

In streptozotocin-induced diabetic rats, Haidari et al. investigated the impact of GTE on body weight, serum glucose levels, and lipids [106]. Administration of GTE (200 mg/kg) led to reduction in body weight loss and a substantial decrease in serum glucose and total cholesterol levels, with no significant effect on serum triglyceride, LDL cholesterol, and HDL cholesterol levels.

Green tea’s ability to reduce glucose and the processes at play in diabetic rodents were studied [107]. Utilizing a test for oral glucose tolerance on T1D rats and T2D KK-Ay mice, drinking green tea was found to lower blood glucose and improve glucose intolerance. Additionally, in both animals, GT decreased the levels of plasma fructosamine and glycated hemoglobin, stimulating GLUT4 translocation and muscle glucose uptake in both animals.

Using a T2D mouse model that combines a high-fat diet and low-dosage streptozotocin (STZ), Tang et al. conducted an in-depth comparison between green tea extracts (GTEs) and black tea extracts (BTEs), both of which have undergone thorough chemical characterization by HPLC [108]. The findings showed that GTE significantly reduced blood glucose levels and improved glucose intolerance while reducing body weight. Additionally, GTE reversed the diabetic liver’s morphological degeneration.

Epigallocatechin gallate (EGCG) and epigallocatechin (EGC), two polyphenol components, were investigated and assessed by Snoussi et al. in a green tea decoction with standard green tea preparations [109]. Furthermore, in a Ussing chamber, *ex vivo* with isolated jejunal loops, and *in vivo* with glucose tolerance tests, the effects of acute (30 min) or chronic (6 weeks) oral administration of green tea decoction (GTD) on intestinal glucose absorption were investigated. The most phenolic chemicals were present in GTD after it had been cooked for 15 min. Acute GTD treatment suppressed SGLT-1 activity, elevated GLUT2 activity, and enhanced glucose tolerance. A similar effect was observed for the phenolic compounds (2/3 EGCG+1/3 EGC). Chronic treatment with GTD enhanced glucose tolerance while reducing body weight and triglyceride and cholesterol levels. Six weeks of treatment with GTD considerably reduced jejunal SGLT-1 and increased GLUT2 mRNA levels while elevating GLUT4 mRNA levels in adipose tissue. These findings indicate that GTD modulates intestinal regulation of glucose digestion and increases adipose GLUT4, offering new information on its potentials in the maintenance of glucose homeostasis in diabetes.

Wu et al. investigated the effect of longtime consumption GT on glucose metabolism and insulin sensitivity in Sprague–Dawley rats [110]. Following 12 weeks of supplementation, GT led to significant decrease in blood glucose, insulin, triglyceride, and free fatty acid levels. GT also enhanced insulin-stimulated glucose uptake and insulin binding in adipocytes. Furthermore, polyphenols extracted from GT enhanced basal and insulin-mediated adipocyte glucose uptake *in vitro*.

In an obese diabetic mouse model that exhibited early important clinical symptoms of non-insulin-dependent diabetes mellitus, Wein et al. investigated the antidiabetic, antiadipogenic, and anti-inflammatory properties of GTE at nonpharmacological dosages [111]. A high dose of GTE fed to female db/db mice led to a transient decrease in blood glucose levels, with concomitant anti-inflammatory activities and no adiponectin-inducing or antiadipogenic effects.

According to Xu et al., consuming green tea can enhance cognitive impairment and lower the prevalence of neurodegenerative disorders [112]. Their findings showed that GT enhances cognitive performance in diabetic rats and prevents hippocampus neuronal death via blocking the JNK/MLCK pathway, thereby offering fresh perspectives on the neuroprotective properties of green tea.

Kang et al. investigated whether green tea that has been fermented with Aquilariae Lignum (fGT) on mice with T2D exhibits a higher antidiabetic impact than unfermented GT [113]. Body mass index (BMI), adiponectin level, fecal excretion, exocrine pancreatic zymogen and serum leptin levels, granules, and periovarian fat level were measured to determine the anti-obesogenic effect of GT in T2D. fGT exhibited potent anti-obesity, anti-hypoglycemic, anti-hyperlipidemic, and antioxidant activities. In comparison to GT, fGT had stronger antidiabetic benefits, thereby indicating that fGT is a strong and promising novel treatment for T2D.

Qu et al. demonstrated how green tea extracts affected the intestinal Na^+^/K^+^-ATPase in T1D and T2D mice [114]. The results reveal that GTE significantly reduced blood glucose levels. While they are even normal in T2D mice, the Na^+^/K^+^-ATPase activities in the gut linked to the absorption of glucose were elevated in T1D mice. These activities were suppressed by GTE, indicating an anti-postprandial hyperglycemia effect.

In a fructose-fed rat model, Wu et al. investigated the effect of GT consumption on high blood pressure, insulin resistance, and GLUT1 and GLUT4 levels in adipose tissues [115]. The study consisted of rats fed on chow and water (control group), a high-fructose diet and water (fructose group), or the same high-fructose diet but with green tea (0.5 g of lyophilized green tea powder dissolved in 100 mL of deionized distilled water) rather than water (fructose/green tea group). Following a 12-week research period, there was an elevation in FBG and insulin levels, high blood pressure in the fructose group, as well as concomitant alterations in insulin-stimulated glucose uptake, insulin binding, and GLUT4 expression in their adipocytes. These metabolic alterations were significantly reversed in the fructose/green tea group, thus indicating the ability of GT to mitigate metabolic defects by improving insulin sensitivity and GLUT4 expression.

#### 6.2.2. Clinical Trials

Takahashi et al. investigated the impact of scheduling acute catechin-rich GT on postprandial metabolism of glucose in young men [116]. Four experiments consisting of (1) a morning placebo experiment (MP trial), (2) evening placebo trials (EP trial, 17:00 h), (3) morning catechin-rich green tea trials (MGT trial, 9:00 h), and (4) evening catechin-rich green tea trials (17:00 h) were conducted. Blood was collected from subjects during fasting, and at random intervals of 30, 60, 120, and 180 min after eating. The MGT trials had substantially higher levels of glucose than the MP trials at 120 min (*p* = 0.031) and 180 min (*p* = 0.013) after meal ingestion. Furthermore, glucose levels were considerably reduced in the EGT trials than the EP trials at 60 min (*p* = 0.014). Additionally, in both the morning and evening trials, the levels of insulinotropic peptides were considerably reduced in the GT trials than in the placebo trials at both the 30 and 60 min points after meal intake (morning: *p* = 0.010; evening: *p* = 0.006). Thus, indicating that evening consumption of catechin-rich green tea suppresses postprandial hyperglycemia.

Huang et al. investigated the relationships between GT consumption on impaired tolerance to glucose (IGT) and diminished fasting glucose (IFG) [117]. The research consisted of 4808 healthy individuals. Consumption of green tea was linked to a reduced likelihood of IFG. The adjusted odds ratios for IFG for green tea consumption of <1, 1–15, 16–30, and >30 cups per week were 1.0, 0.42 (95% confidence interval (CI) 0.27–0.65), 0.23 (95% CI 0.12–0.46), and 0.41 (95% CI 0.17–0.93), respectively. Individuals with lower odds ratios for IFG were those who drank 16 to 30 cups of GT per week, thus indicating that consumption of 16 to 30 cups of GT per week is associated with lower risk of developing T2D.

Hua et al. also investigated how obese individuals with T2D responded to a daily dose of 856 mg of EGCG from decaffeinated green tea extract (GTE) [118]. For 16 weeks, individuals with T2D (BMI > 25 kg/m^2^) received 500 mg of decaffeinated GT. No measurable variable showed a statistically significant variance between the decaffeinated GTE group and the group receiving placebo. In comparison to baseline, there was a statistically important within-group HbA1C drop of 0.4% following GTE treatment. There were substantial decreases in the circumference of the waist (WC), HOMA-IR index, and insulin level, as well as a substantial rise in ghrelin level in the GTE group. Ghrelin levels significantly increased among those in the placebo group as compared within-group. The decaffeinated GTE and placebo groups showed no statistically noteworthy distinctions in any of the investigated variables, yet some significantly interesting within-group variations were recorded.

**Table 2:** Antidiabetic properties of green tea.

Cell Lines/Tissue/Animal Model/Human	Study	Treatment	Antidiabetic Mechanism	Reference
Human hepatoma cells (HepG2), mouse myoblasts (C_2_C1_2_), and mouse fibroblasts (3T3-L1)	*In vitro*	Cells incubated with 0.01, 0.1, 1, and 10 M concentrations of urolithin green tea flavonoid for 24 h.	Green tea phytoconstituents significantly increased glucose uptake in all tested cells. Reduced lipid accumulation in HepG2 and 3T3-L1 (adipocyte) cells. Inhibited α-amylase and α-glucosidase digestive enzymes.	[91]
Intestinal glucose transport model in Caco-2 cells		Caco-2 cells seeded into 12-well plates were treated with 500 μL green tea crude and ethanol extracts diluted with PBS.	Treatment with tea extract lowered the % of glucose transported from cells’ apical media end to the basolateral media side.	[92]
Glucose transport in Caco-2 cell model		Cultured cells were incubated with gelatinized wheat starch mixed with 0.02 g green tea extract for 2 h.	Lowered wheat starch glucose transported via cells.	[94]
		Cells seeded at 3.0 × 10^5^ cells/well were incubated with 500 µL glucose-containing solution with green tea extract.	Tea extract weakly inhibited pancreatic α-amylase and α-glucosidase enzymes. Decreased glucose transport through Caco-2 cell monolayer.	[95]
Insulin amyloid aggregation assay		Green tea epigallocatechin gallate at 200 pM to 1 mM in insulin solution was subjected to aggregate-inducing conditions.	Presence of the tea compound lowered insulin amyloid fibril formation.	[119]
Wistar rat excised jejunal segments and brush border membrane vesicles from the rabbit’s small intestine	*Ex vivo*	Animal tissues were incubated in a solution containing green tea epicatechin gallate for 12 h.	Tea compounds inhibit SGLT-1 and significantly decrease glucose uptake in the isolated tissues.	[93]
STZ-induced diabetic male Wistar rats	*In vivo*	100 mg/kg BW green tea infusion administered once daily for 42 days.	Green tea infusion considerably reduced the amount of kidney glycogen granules. Increased numbers of renal cortex cells without DNA damage. Regulated kidney Na^+^/K^+^ ATPase concentration to control level.	[97]
		*Ad libitum* administration of 0.1% green tea extract for 8 weeks.	Reduced serum glucose level. Slightly increased liver and kidney catalase activity. Decreased the expression of angiotensin II AT1 receptor in liver and kidney tissues.	[103]
		Animals were given 1.5% (w/v) green tea aqueous extract as a drinking solution for 76 days.	The tea extract lowered levels of serum biomarkers of hyperglycemia-induced hepatic damage. Increased antioxidant capacity in the liver.	[104]
		Oral administration of 100 mg/kg BW and 200 mg/kg BW green tea for 4 weeks.	Extract significantly reduced serum glucose and cholesterol levels. Slightly improved body weight in diabetic animals.	[106]
		Rats were injected intragastrically with 5 and 10 mL/kg/day of green tea concentrated for 8 weeks.	Tea extract-suppressed hyperglycemia induces hippocampus neuron apoptosis by decreasing Bax, pJNK, and myosin light chain kinase protein expression.	[112]
High-fat diet-fed male Sprague–Dawley rats		Infusion of 1 green tea bag per 200 mL water given as 100% fluid intake for 27 weeks.	Green tea improved glucose tolerance. Increased liver expression of genes involved in fatty acid synthesis and oxidation. Lowered expression of genes linked with adipocyte differentiation in peripheral tissue.	[96]
High-fat diet and streptozotocin-induced diabetic rats		75, 150, and 300 mg/kg BW green tea given daily intragastrically for 30 days.	Green tea extract increased serum insulin level. Reduced serum glycated hemoglobin concentration. Regulated liver metabolic enzymes’ activity to normal levels.	[98]
High-fat diet-induced obese mouse model		Green tea extract 500 mg/kg BW given once a day for 12 weeks.	Tea lowered miR-335 gene expression in adipocytes. Reduced serum proinflammatory cytokine levels. Improved expression of metabolic genes in white adipose tissue. Enhanced adipocyte insulin sensitivity.	[99]
High-fat diet-induced obese wild-type (C57Bl/6) and adiponectin knockout mouse models		500 mg/kg BW orally daily dose of green tea extract for 12 weeks.	Tea lowered adipocyte size and body weight gain in wild-type animals. Decreased insulin resistance in both wild-type and adiponectin knockout mice. Enhanced browning of subcutaneous white adipose tissue in C57Bl/6 mice. Increased thermogenesis in the brown adipose tissue of wild-type animals.	[100]
High-fat diet-induced obesity model		Mice were orally administered 75 mg/kg BW green tea epigallocatechin gallate compound.	Tea active ingredient significantly improved oral glucose tolerance.	[96]
High-fat diet-induced obesity model STZ-induced diabetes in high-fat diet-fed CD-1 mice		Male rats were gavaged 0.5 mL/100 g BW green tea decoction for 6 weeks.	The tea decoction increased GLUT-2 gene expression and activities in rat jejunum. Increased GLUT4 gene expression in adipose tissue. Significantly lowered SGLT-1 activities in the jejunum.	[109]
		Female animals were administered 0.01% green tea via drinking water for 12 weeks.	Green tea suppressed animal body weight. Lowered both fasting and non-fasting blood glucose levels. Improved glucose tolerance and enhanced plasma insulin. Mitigated lipid-induced hepatic damage.	[108]
STZ-induced diabetes in high-fat diet-fed albino rats		Animals had 75 mg/kg BW, 150 mg/kg BW, and 300 mg/kg BW green tea extract for 30 days.	Tea treatment lowered glycated hemoglobin in the blood. Improved hepatic and muscle glycogen content. Enhanced serum insulin concentration. Normalized altered hepatic enzyme activities.	[98]
STZ-induced type 2 diabetes in high-fat diet-fed mice		Animals received 300 mg/kg BW green tea intragastrically for 4 weeks.	Treatment with tea extract suppressed Na^+^/K^+^-ATPase activity and protein expression in the small intestine of diabetic animals.	[114]
High-fructose diet-fed rat model		Fresh solution of 0.5 g/100 mL lyophilized green tea was given daily for 12 weeks.	Tea sample increased insulin binding to adipocytes. Increased adipocyte GLUT-1 and GLUT-4 protein expression.	[115]
High-fructose diet-induced insulin resistance in rats		Wistar rats fed synthetic fructose-rich diet (200 g/kg) supplemented with 1 g/kg or 2 g/kg green tea sample for 6 weeks.	Tea presence in food slightly reduced animal weight. Lowered fasting blood glucose. Reduced serum triacylglycerol concentration.	[56]
STZ-induced type 1 and type 2 diabetes in rat and mice models		Animals received green tea orally for 56 days.	Green tea enhanced GLUT4 protein expression in rat muscle. Decreased serum triglycerides.	[96]
Sucrose-induced hyperglycemia		Rats were given 0.5 g/kg BW green tea aqueous extract after 2 g/kg BW sucrose loading.	Tea extracts inhibited α-glucosidase enzyme. Lowered plasma glucose concentration.	[94]
Alloxan-induced diabetic male Balb/C mice		Animals were gavaged 300, 600, and 1200 mg/kg BW doses of green tea extract for 14 days.	Treatment with tea lowered blood glucose and maintained liver ultrastructural architecture.	[102]
db/db mouse model of diabetes		Animals fed for 10 weeks with a diet supplemented with 10 g/kg epigallocatechin gallate-rich green tea.	Green tea significantly lowered fasting blood glucose and body weight. Increased pancreatic islet β-cell population. Elevated plasma insulin level.	[101]
Type 2 diabetes model in db/db mice		Oral administration of 100, 200, and 400 mg/kg fermented green tea once daily for 84 days.	Tea treatment decreased serum leptin and adiponectin concentrations. Lowered serum total cholesterol and triglyceride levels. Increased pancreatic insulin immunolabelled cells. Increased hepatic antioxidant capacity.	[113]
Normal male Sprague–Dawley rats		Animals were provided 0.5 g/100 mL of fresh green tea drink daily for 12 weeks.	Green tea supplement lowered plasma glucose and insulin levels. Increased insulin binding and glucose uptake in adipocytes.	[110]
Normal female db/db mice		Animals were administered 0.1 g/kg and 1 g/kg green tea extract-enriched diet daily for 28 days.	Green tea lowered soluble intercellular adhesion molecule-1 (sICAM-1) in the plasma of animal subjects.	[111]
Hyperglycemia-induced test meal in postmenopausal women		Females took green tea beverages containing 615 mg/350 mL of total catechins with breakfast daily for 4 weeks.	Tea raised thioredoxin concentration in serum 6 h after administration. Reduced postprandial blood glucose.	[116]
Obese patients with type 2 diabetes		Individuals with type 2 diabetes (BMI > 25 kg/m^2^) received 500 mg of decaffeinated green tea table thrice daily for 16 weeks.	Treatment significantly reduced plasma glycated hemoglobin. Elevated blood ghrelin concentration.	[118]
Healthy human subjects	Clinical	Participants took between 1 and above 30 cups of tea green tea for 1 week (1 = 150 mL tea infusion).	The tea reduced fasting plasma glucose.	[117]
Healthy male subjects		Human subjects consumed 615 mg/350 mL) catechin-rich beverage with a test meal within 10–15 min.	The tea considerably lowered postprandial blood glucose level. Reduced the plasma concentration in glucose-dependent insulinotropic polypeptide.	[116]

### 6.3. Oolong Tea

The antidiabetic properties of oolong tea are summarized in Table 3.

#### 6.3.1. *In Vitro* Studies

The properties of black, green, and oolong teas and their constituent parts to increase insulin were identified by Anderson and Polansky [120]. In an epididymal fat cell experiment, oolong tea consumption was found to boost the activity of insulin >15-fold *in vitro*. Using a high-performance liquid chromatography separation of tea extracts using a Waters SymmetryPrep C18 column, it was discovered that epigallocatechin gallate was primarily responsible for the insulin-potentiating activity of oolong tea. Epigallocatechin gallate, epicatechin gallate, tannins, and theaflavins all showed a greater insulin-enhancing effect than the other known tea constituents. Catechin, epicatechin, and caffeine all showed negligible insulin-enhancing properties. The insulin-potentiating activity was reduced by 90% when 50 g of milk was added per cup, compared to a reduction of 5 g when 2% milk was added. Soy milk and non-dairy creamers also reduced the insulin-enhancing action. The results presented show that tea’s main active component, epigallocatechin gallate, has an *in vitro* insulin-enhancing effect.

Furuyashiki et al. explored into how different tea extracts affected the transportation of glucose in 3T3-L1 adipocytes [121]. Despite the influence of insulin stimuli, 3-O-methylglucose (3-OMG) absorption was inhibited by oolong tea extracts. By suppressing GLUT4 translocation, oolong tea extract altered the glucose transport mechanism. According to these findings, oolong tea extracts alter the glucose transport system via lowering GLUT4 release.

Fei et al. investigated pancreatic α-amylase inhibition, enzyme kinetics, ultraviolet (UV) absorption spectrum, and fluorescence spectrum in order to determine the effect and potential mechanisms of oolong tea polyphenols, (−)-epigallocatechin gallate (EGCG), and (−)-epigallocatechin 3-O-(3-O-methyl) gallate (EGCG3′′Me) [122]. The findings demonstrated that oolong tea polyphenols, EGCG, and EGCG3′′Me displayed inhibitory activities against α-amylase, with their half-inhibitory concentration (IC_50_) values being 0.375, 0.350, and 0.572 mg/mL, respectively. Lineweaver–Burk double reciprocal plot revealed that the inhibitory activities of EGCG and the oolong tea polyphenols were competitive. However, EGCG3′′Me was in an ineffective form. Oolong tea polyphenols, EGCG, and EGCG3′′Me all caused a red-shift in UV absorbance and a quenching of α-amylase’s fluorescence, which may indicate alterations in the enzyme’s conformation. These variations in inhibitory properties may be attributed to the structural changes between EGCG and EGCG3′′Me (replacement of the hydroxyl group with the methoxy group at position 3 in the D ring of EGCG to generate EGCG3′′Me).

According to Rujanapun et al.’s research [123], a special Thai oolong tea has an interesting chemical profile and exhibits *in vitro* antidiabetic properties. Oolong tea steamed with ginger (*Zingiber officinale*), celery (*Anathallis graveolens* L.), and lemongrass (*Cymbopogon citratus*) is called eternity tea (EN), while peaceful rest (PR) tea is made of oolong tea leaves steamed with wild betel leaf bush leaves (*Piper sarmentosum*), Indian gooseberry (*Phyllanthus emblica*), and Turkey berry (*Solanum torvum*). PR extract showed the highest level of biological activities in *in vitro*, including antioxidant, anti-inflammatory, antiadipogenic, enzyme inhibition, and glucose absorption and intake. The PR and EN extracts’ UHPLC-QTOF-MS/MS profiles revealed chemical profiles distinct from those of oolong tea. For example, piperettine I was discovered in PR, but gingerdiol and gingerol were detected in EN. It is thus apparent that, between the three tea extracts, PR’s added components contributed to better biological activities than those of oolong and EN. It is also significant to note that PR extract suppressed metformin action (*p* < 0.05), as well as glucose absorption and utilization by adipocytes and skeletal muscle at doses of 500 and 100 μg/mL, respectively. The results of this investigation confirm that oolong tea steamed with *P. sarmentosum*, *P. emblica*, and *S. torvum* had higher antidiabetic properties.

#### 6.3.2. *In Vivo* Studies

Imaga and Hunga conducted an analysis of oolong tea’s phytochemical makeup, nutritional composition, antioxidant characteristics, and impact on specific rat organs and tissues [124]. The tea extract was discovered to contain several phytochemicals, with significant concentrations of phenol (157.84 μg GAE/mg), flavonoids (158.15 μg GAE/mg), and tannins (343.3 μg GAE/mg). Oolong tea was made up of 45% crude fiber and 4% moisture. High levels of total antioxidant and DPPH free radical scavenging activities were demonstrated by *in vitro* antioxidant assays, with 25% concentration showing the maximum activity in both cases. The lipid level of the experiment’s rats compared to the control group decreased after daily administration of the tea extract to rats, with concomitantly reduced blood glucose levels compared to the control group. Liver function test demonstrated reduced liver enzyme levels, indicating enhanced functioning of the liver, which can be attributed to the antioxidant properties of the extract. In conclusion, oolong had a favorable response *in vivo* and showed potentials for application as a hypoglycemic and hypocholesterolemic agent.

Administration of oolong tea to male ICR mice led to decrease in the weight of white adipose tissue and the amount of total cholesterol [125]. It also led to phosphorylation of PI3K and AMPK while concomitantly enhancing GLUT4 translocation.

The effect of oolong tea on the translocation of GLUT4 and associated signaling cascades was investigated in skeletal muscles of C57BL/6J mice and ICR mice [126]. Oolong tea improved glucose tolerance by activating the PI3K/Akt- and AMPK-dependent signaling pathways. This led to the translocation of GLUT4 and improved expression of the insulin receptor, thereby indicating that oolong tea can protect against the development of diabetes.

Yasui and his team investigated how oolong tea consumption affects glucogenic genes in mouse liver [127]. Four-week consumption of oolong tea decreased hepatic expression of PEPCK, G6Pase, and hepatocyte nuclear factor (HNF4α) as well. According to the results, the activity of these genes was suppressed when rat hepatoma H4IIE cells were treated with oolong tea. Additionally shown was that PEPCK and HNF4α decreased protein expression.

#### 6.3.3. Clinical Trials

In Miaoli, Taiwan, Hosoda et al. investigated the effectiveness of oolong tea in decreasing plasma glucose in patients with T2D [128]. The study had twenty unconfined patients with T2D who took high-in-glucose medications as directed. In a randomized crossover layout, participants drank either 1500 mL of oolong tea or water daily for a period of thirty days. Prior to therapies, tea consumption was restricted for a period of fourteen days. Plasma levels of fructosamine and glucose were significantly reduced by oolong tea but not by water, thereby indicating oolong tea as a potential oral hypoglycemic agent for the management of T2D.

**Table 3:** Antidiabetic properties of oolong tea.

Cell Lines/Tissue/Animal Model/Human	Study	Treatment	Antidiabetic Mechanism	Reference
Rat epididymal adipocytes	*In vitro*	Adipocytes were incubated with 0.43 *í*Ci of glucose, 72 *í*g of glucose, and insulin and/or tea extract in a final reaction volume of 2 mL of Krebs-Ringer phosphate (pH 7.4) for 1 h.	Oolong tea increased insulin sensitivity.	[120]
Murine 3T3-L1 preadipocytes		Mature 3T3-L1 adipocytes were treated with tea extract in 1 mL of Krebs-Ringer phosphate–HEPES buffer and insulin stimulation was performed for 15 min at 100 nM, incubated with 6.5 mM 3-OMG for 30 s.	Oolong tea extract modulated the glucose transport system through translocation of GLUT4.	[121]
3T3-L1 preadipocytes and L6 myoblast cells		3T3-L1 and L6 cells were initially cultured with extract and glucose for 24 h, then incubated with glucose and Krebs-Ringer bicarbonate buffer for 1 h, respectively.	Oolong tea fortified with Indian gooseberry markedly inhibited α-amylase and α-glucosidase. It enhanced glucose consumption on adipocyte and glucose uptake on L6 muscle cells.	[123]
Digestive enzyme inhibition		α-amylase inhibitory assay with oolong tea polyphenols.	Oolong tea polyphenols inhibited α-amylase in a competitive pattern.	[122]
Rat hepatoma H4IIE cells and male BALB/c mice	*In vivo*	4 h incubation of cultured H4IIE cells with tea extracts Oral administration of tea *ad libitum* for 4 weeks.	Oolong tea significantly repressed the expression of G6Pase and PEPCK in H4IIE cells. Likewise, the tea reduced the expression of G6Pase, PEPCK, and hepatocyte nuclear factor-4α (HNF-4α) in the mice’s livers.	[127]
Male ICR mice		Daily consumption of tea extract for 1 week.	Fermented oolong tea extract markedly decreased white adipose tissue weight and the total cholesterol level. Its facilitated translocation of GLUT 4 through the stimulation of phosphoinositide 3-kinase (PI3K) and 5′AMP-activated protein kinase (AMPK) phosphorylation.	[125]
Male C57BL/6J mice and male ICR mice		C57BL/6J mice were fed high-fat diet with tea extract for 14 weeks, while ICR mice were fed commercial chow with tea extract for 7 days.	Oolong tea activates PI3K/Akt- and AMPK-dependent signaling pathways to induce GLUT4 translocation. Improves glucose tolerance by increasing the expression of insulin receptor.	[125]
Healthy female Wistar albino rats		Daily oral administration of 1.2 mL oolong tea extract to rats weighing 175 g while those weighing 200 g were given 1.3 mL for 3 weeks.	Oolong tea decreased lipid profile and glucose levels in rats. Improved liver function enzymes and antioxidant capacity in rats.	[124]
Human subjects with type 2 diabetes	Clinical	1.5 l daily consumption of tea for 30 days.	Oolong tea significantly decreased glucose and fructosamine concentrations.	[128]

### 6.4. Black Tea

The antidiabetic properties of black tea are summarized in Table 4.

#### 6.4.1. *In Vitro* Studies

Ma et al. investigated the possibility that black tea (BT) may contain strong PTP1B tyrosine phosphatase inhibitors [129]. A common tyrosine phosphatase known as PTP1B has been identified as a therapeutic target for the treatment of obesity and diabetes [130]. The aqueous extracts of the tea showed strong PTP1B inhibitory effects. It was further confirmed that oxidation by tyrosinases transformed tea catechins into strong inhibitors of PTP1B. The extracts enhanced tyrosine phosphorylation of cellular proteins when administered to cultured cells.

Nagano et al. investigated whether GLUT4 translocation in L6 myotubes is facilitated by black tea polyphenols (BTPs) [131]. In L6 myotubes, BTPs facilitated GLUT4 translocation and glucose absorption. BTPs increased the phosphorylation of Akt Thr308, Akt substrate 160, atypical PKC, and AMP-activated protein kinase (AMPK) but did not stimulate the phosphorylation of Akt Ser473. GSK-3 was inactivated by BTP, leading to increased glycogen storage. One of the main ingredients in black tea, theaflavin, also stimulated glucose absorption and GLUT4 translocation, as observed with BTPs in L6 myotubes. These findings demonstrate that BTPs promote GLUT4 translocation and glycogen synthesis in skeletal muscles via activation of PI3K- and AMPK-dependent pathways, with theaflavin playing an influential role.

Striege et al. investigated the probable mode of effect and biological activity of BT and black tea pomace for T2D management via inhibition of carbohydrate-hydrolyzing enzymes [132]. Hot water was used to extract the leaves of black tea (WBT), and 70% acetone was used to extract the black tea pomace (AOBT). Low molecular weight phenolic-enriched fraction (LMW), high molecular weight-enriched fraction (HMW), and hydrophobic fraction (HBBT) were further obtained from WBT. The LMW and HMW fractions had phenolic contents of 1.42 and 2.66 mg/mL, respectively. HMW exhibited the highest inhibitory activity on α-glucosidase, while HBBT was the highest for α-amylase. AOBT exhibited considerable glucosidase inhibitory activity, although its inhibitory effect on α-amylase was less potent. The study highlights that high molecular weight phenolics influence the inhibitory activities of BT in carbohydrate-hydrolyzing enzymes.

The benefits of BT and its constituents for boosting insulin were reported by Anderson and Polansky [120]. In a test using epididymal fat cells, it was discovered that drinking BT increased insulin activity by >15-fold *in vitro*. This activity was influenced by the presences of theaflavins, tannins, and EGCG, which have been reported for their enhancement of insulin sensitivity. The insulin-enhancing effects of epicatechin, caffeine, and catechin were minimal. The insulin-potentiating activity was reduced by one-third following the addition of 5 g of 2% milk per cup and by 90% when 50 g of milk was added. Soy milk and non-dairy creamers also reduced the insulin-enhancing activity. These findings demonstrate insulin-enhancing effect of BT, with epigallocatechin gallate being the main active component.

Xiao et al. assessed the phytochemical, antioxidant, and antidiabetic properties of BT [133]. *In vitro* study with concentrated infusions of BT revealed considerable free radical scavenging capabilities. BT increased muscle glucose uptake while inhibiting intestinal glucose absorption and α-amylase activity. The presence of hydroxycaffeic acid, l-threonate, caffeine, vanillic acid, n-acetylvaline, and spinacetin 3-glucoside in *C. sinensis* was identified following LC-MS profiling.

#### 6.4.2. *In Vivo* Studies

Abeywickrama et al. investigated Sri Lankan BOPF-grade tea’s ability to decrease blood glucose levels as well as its medicinal value in relation to agroclimatic heights [134]. High, mid, and low agroclimatic elevations in Sri Lanka were used to obtain unblended orthodox BOPF-grade tea samples. The tea infusion (BTI) (60, 120, and 480 mg/mL) was orally administered to STZ-induced diabetic rats. BTI showed rapid onset, dose-dependent, substantial, and potent hypoglycemic and antihyperglycemic activities. Despite variations in the content of phytoconstituents, these impacts were not affected by agroclimatic altitude. BTI suppressed α-glucosidase and α-amylase activities and intestinal glucose absorption. BTI also improved *in vivo* antioxidant properties, insulin mimetic activity, and insulin sensitivity. Thus, Sri Lankan BT of any agroclimatic height and BOPF grade possesses hypoglycemic and antihyperglycemic properties that may be utilized in controlling blood glucose levels.

In a T2D mouse model, Tang et al. investigated the antidiabetic effect of chemically characterized BT extracts [108]. The research investigated the antidiabetic properties of black tea extracts (BTEs) that have undergone thorough chemical characterization by HPLC in a T2D mouse model using low-dose STZ and HFD. The findings showed that BTE significantly reduced blood glucose levels and improved glucose tolerance and insulin secretion while improving the liver’s histological degeneration.

Atiku et al. investigated explored how Turkish and Ceylon BT affected metabolic syndrome growth in diabetic rats [135]. Diabetic rats were orally administered 2.8 mg/kg BW of the teas. The teas significantly reduced the rats’ fasting blood glucose (FBS) levels and improved serum lipid profiles, suggesting their hypoglycemic and hypocholesterolemic characteristics.

In STZ-induced diabetic rats, Ramalingam et al. investigated the effect of BTE on carbohydrate metabolic enzymes, blood glucose, tricarboxylic enzymes, plasma insulin and HbA1C levels [136]. Diabetic rats received doses of 25, 50, and 100 mg/kg BW of BTE daily for 30 days. BTE caused significant reduction in blood glucose and HbA1C levels and improved insulin secretion while improving carbohydrate metabolism, with the best activity achieved at a dose of 100 mg/kg BW.

In KK-AY/TaJcl diabetic mice, Shoji and Nakashima investigated the effect of powdered African black tea extract on blood glucose level [137]. Administration of BTE to KK-AqTaJcl mice for 3 months led to a significant reduction in blood glucose level with concomitant improvement in serum lipid profile and insulin secretion. It also improved postprandial blood glucose levels and morphologies of the liver, spleen, kidney, stomach, heart, and pancreas.

Huang and Lin investigated the effect of four distinct tea leaves on male Wistar rats’ blood lipid and leptin levels over the course of a 12-week period [138]. The findings demonstrated that in comparison to the control group, a fructose-rich meal significantly increased serum triacylglycerols, cholesterol, insulin, and leptin concentrations. These levels were significantly reversed following 12 weeks’ consumption of BT. In comparison to rats administered fructose only, rats treated with BT had reduced epididymal adipose tissue weights, with concomitant reduced hepatic levels of FAS mRNA protein and increased AMPK phosphorylation. These results indicate that BT contributes to reduced hyperlipidemia and hyperglycemia.

Akaln et al. employed STZ-induced diabetic mice fed a high-fat diet (HFD) to investigate the antidiabetic activities of BT polysaccharides (BTPSs) [139]. Oral administration of BTPS (400 mg/kg) to STZ-HFD induced T2D C57BL/6J mice for 6 weeks led to a significant decrease in FBG level with improved insulin sensitivity, thus indicating that BTPS can serve as a dietary supplement to manage T2D without posing any possible health risks.

Imada and Ashida studied the effect of BT on overweight and diabetes in KK-Ay diabetic mice [140]. Mice received either water or BT while being fed. The tea prevented increases in plasma glucose and insulin levels compared to the water-only group. It also reduced plasma lipid levels and the weight of white adipose tissue (WAT). In mesenteric white adipose tissue, BT decreased gene expression of the inflammatory cytokines, TNF-α, IL-6, and MCP-1, which evoke insulin resistance, while enhancing the activities of lipid-metabolizing enzymes such as acetyl-CoA carboxylase (ACC) and carnitine palmitoyltransferase-1 (CPT-1). These findings suggest that BT can improve lipid metabolism and reduce hyperglycemia by downregulating the expression of proinflammatory cytokines in WAT.

In alloxan-induced diabetic mice, Rohdiana and team investigated the antidiabetic effects of first-grade orthodox black tea [141]. In comparison to distilled water, administration of the tea infusion showed a superior reduction in blood glucose level.

Male C57BL/6J mice received a diet high in fat comprising 29% lard as well as black tea *ad libitum* for 14 weeks as part of Nishiumi’s experiment on the preventative effects of BT on diabetes and insulin resistance [142], with the results showing reduced dietary-induced white adipose tissue formation and increased body weight. The diet also enhanced glucose absorption and GLUT4 levels in muscle while improving glucose tolerance. Continuous dietary fat consumption decreased levels of GLUT4, AMPK, and insulin receptor subunit in muscles but was reversed following the administration of BT. The findings strongly imply that drinking BT can mitigate the effects of a high-fat diet on diabetes and improve insulin sensitivity by maintaining GLUT4 levels.

Qu et al. demonstrated how black tea extracts affected intestinal Na^+^/K^+^-ATPase activity in T1D [114]. The results of this study suggest that BTE significantly reduced blood glucose levels in STZ-induced T1D and STZ-HFD induced T2D mice. They demonstrated that BTE can reduce intestinal Na^+^/K^+^-ATPase disruption in T1D, which helps to reduce postprandial hyperglycemia. This activity was attributed to the tea’s primary active ingredients such as theaflavins and theaflavine-3,3′-digallate. This indicates that the postprandial–hypoglycemic properties of black tea in T1D are brought about by the tea’s ability to inhibit the activity of intestinal Na^+^/K^+^-ATPase.

Using a mouse model with an established diet high in fat and streptozotocin (STZ)-induced hyperglycemia, Shang et al. evaluated the hypoglycemic properties and probable mechanisms for aqueous extracts of regular BT and selenium-enriched black tea (Se-BT) [143]. Both BT and Se-BT were discovered as potent inhibitors of α-glucosidase activity. Both teas had a stimulatory effect on the PI3K/Akt signaling pathway, thus reducing insulin resistance and diabetes. They also improved the gut flora.

#### 6.4.3. Clinical Trials

Bryans and team investigated how drinking BT affected healthy individual’s postprandial plasma glucose and insulin levels in response to an oral glucose load [144]. Sixteen healthy, fasting volunteers ingested 75 g of glucose in one of four combinations, control, 250 mL of water plus 0.052 g of caffeine (positive control), 250 mL of water plus 1.0 g or 3.0 g of instant black tea, or 250 mL of water plus all four combinations. Blood samples were taken at 30 min intervals for 150 min of consumption. Plasma glucose levels in reaction to the beverages were identical after 60 min but were considerably lower at 120 min after consumption of 1.0 g tea drink compared to the control and caffeinated drinks. At 90 min, drinking tea resulted in higher levels of insulin compared to control and caffeine drinks, and at 150 min compared to drinking caffeine alone. With a concomitant rise in glucose, the 1.0 g tea beverage decreased late-phase blood glucose sensitivity, thus demonstrating the ability of BT to cause an increase in insulin secretion in response to increased glucose consumption, thereby leading to reduced blood glucose level.

Butacnum et al. investigated how drinking BT affected patients with normal and prediabetic blood glucose levels and insulin responses after sucrose loading [145]. This study was a crossover phenomenon, double-blind, randomized trial. A solution containing sucrose with a low dose (110 mg BTPP) of black tea beverage (BTB), a high dose (220 mg BTPP) of BTB, or a placebo beverage (0 mg BTPP) was given to twenty-four volunteers, both male and female, aged 20 to 60, who were healthy and prediabetic. In order to determine the blood glucose and insulin levels, blood samples were taken at 0, 30, 60, 90, and 120 min after the start of tea consumption. In comparison to placebo, the low-dose and high-dose BTPP significantly reduced incremental blood glucose area under the curve (AUC) in both normal and prediabetic subjects (T0-60 min 3232 vs. 3295 vs. 3652 mg/min/dL; *p* = 0.016; T0-60 min 2554 vs. 2472 vs. 2888). In terms of blood glucose variations, there was no significant difference between the placebo and BT groups. There were no appreciable variations in side effects across the placebo, low-dose, and high-dose BTPP groups, thus further corroborating the association of BT consumption with reduced postprandial blood glucose levels.

Isono and team investigated how BT consumption affects postprandial blood glucose levels in healthy Japanese individuals [146]. Participants consumed 200 mL of BT, and it led to reduced sucrase, α-amylase, and α-glucosidase activities. Furthermore, 200 mL of BT was further administered to 46 healthy Japanese individuals after consumption of 200 g of cooked rice, leading to a reduced incremental area under the curve (AUC) for glucose and insulin (*p* = 0.024 and *p* = 0.014, respectively) compared to a placebo. These activities were attributed to the identified high molecular weight polyphenols, which included galloyl moieties, thus also corroborating the ability of BT to suppress dietary-induced postprandial rise in blood glucose levels.

Mahmoud et al. investigated how drinking BT affected metabolic biomarkers and inflammation in individuals with T2D [147]. Thirty patients with T2D were randomly assigned to one of two groups: the high-intake (HI) group, which received three cups (600 mL) of BT per day, or the low-intake (LI) group, which received only one cup (200 mL) per day throughout the course of a 12-week period. At baseline and after the tea consumption period, the levels of intracellular cytokines, regulatory T cells (Treg), glycemic profiles, and lipid profiles were assessed. BT consumption was linked with significant variations in HbA1c levels, increased T cells that regulate inflammation (CD3+ CD4+ CD25+ FOXP3), CD3+ CD4+ IL-10+ cells (an immunosuppressive phenotype), decreased proinflammatory CD3+ CD4+ IL-17+ cells, and Th1-associated CD3+ CD4+ IFN-Y+ cells. These findings indicate that the hyperglycemic activity of BT in T2D is associated with its ability to modulate inflammatory cytokine levels.

**Table 4:** Antidiabetic studies of black tea.

Cell Lines/Tissue/Animal Model/Human	Study	Treatment	Antidiabetic Mechanism	Reference
NIH-3T3 cells	*In vitro*	Cultured NIH-3T3 cells were incubated with the tea extracts for 30 min.	Black tea extracts and black tea catechins potently inhibited protein tyrosine phosphatases 1B. The tea extracts induced strong tyrosine phosphorylation of cellular proteins in cultured NIH-3T3 cells.	[129]
L6 myotubes		Cultured L6 cells were incubated with 0.1, 1, or 10 µg/mL of black tea polyphenol (BTP) for 15 min and further treated with 100 nM insulin for 15 min.	BTP promoted GLUT4 translocation through PI3K- and AMPK-dependent pathways and facilitated glucose uptake in L6 cells. Enhanced accumulation of glycogen via inactivating GSK3β.	[131]
Rat epididymal fat cells		0.43 µCi of glucose, 72 µg of glucose, and adipocytes were incubated with insulin and/or tea or its components for 1 h.	Black tea and its active component (epigallocatechin gallate) demonstrated insulin sensitivity-enhancing activity.	[120]
Digestive enzymes inhibition		Carbohydrate-digestive enzyme inhibitory assays with black tea extract and black tea pomace polyphenols (AOBTs).	Both the tea extract and AOBT markedly inhibited α-glucosidase and α-amylase enzymes.	[132]
Healthy rat Psoas muscle and intestinal jejunum		Co-incubation of muscle or intestine with 11.1 mM glucose and different doses of black tea extract for 2 h.	Black tea extract significantly inhibited α-amylase activity and intestinal glucose absorption while elevating muscle glucose uptake.	[133]
STZ-induced diabetes in male albino rats	*In vivo*	480 mg/dL oral daily dose of tea extract for 14 days.	Black tea infusion impaired α-glucosidase and α-amylase while inhibiting intestinal glucose absorption. Showed dose-dependent and significant hypoglycemic and anti-glycemic activities with quick onset. Significantly lowered fasting blood sugar level and increased insulin sensitivity. Exhibited insulinomimetic action.	[134]
		Administration of 25, 50, and 100 mg/kg body weight (BW) black tea extract for 30 days.	Black tea significantly reduced systemic glucose and glycated hemoglobin levels while increasing insulin levels in a dose-dependent manner.	[136]
STZ-induced diabetic mice model		Administration of 100 mg/kg BW black tea extract for 30 days.	Tea extract significantly reduced blood glucose level and glycated hemoglobin and increased insulin levels. Restored the activities of impaired carbohydrate-metabolizing enzymes.	[148]
STZ and high fat-induced type 2 diabetic C57BL/6J male mice		Oral administration of 400 mg/kg BW of black tea polysaccharides (BTPs) for 6 weeks.	BTPs significantly reduced fasting blood sugar level and improved insulin resistance. Suppressed body weight gain.	[139]
STZ-induced T1D and high-fat diet/STZ-induced T2D mice model		Administration for 300 mg/kg BW for 4 weeks.	Black tea exhibited significant hypoglycemic effect by reducing protein expression and the activities of Na^+^/K^+^-ATPase activity.	[114]
High-fat diet/STZ-induced hyperglycemia		Administration of 400 mg/kg black tea extract in drinking water for 5 weeks.	Black tea inhibited α-glucosidase activity. Regulated the expression of mRNA as well as glucose and lipid metabolisms. Alleviated tissue damage and restored intestinal flora dysbiosis. Activated the phosphoinositide-3-kinase/protein kinase B (PI3K/Akt) signaling pathway while alleviating hyperglycemia and insulin resistance.	[143]
		1 mg/mL daily dose of tea extract for 8 weeks.	Black tea extract significantly lowered blood glucose level and ameliorated glucose intolerance. Increased serum insulin levels.	[108]
Fructose-induced metabolic syndrome		Oral administration of 2.8 mg/kg BW of the tea extract for 7 weeks.	The tea extract significantly reduced fasting blood glucose level. Decreased uric acid level. Improved the serum lipid profile in rats.	[135]
		A daily diet of 60% fructose, 36% Purina chow, and 4% black tea leaves for 12 weeks.	Black tea significantly reduced serum triacylglycerol, cholesterol, insulin, leptin, and non-esterified fatty acid concentrations. Markedly reduced hepatic fatty acid synthase (FAS) mRNA level and elevated AMPK phosphorylation.	[138]
KK-A^γ^/TaJcl model of type 2 diabetic mice		Oral administration of 5, 10, or 50 mg/kg of powder formulation of black tea extract dissolved in water for 4 weeks.	Significant suppression of elevated blood glucose on oral glucose tolerance test in 8-week-old KK-Aγ/TaJcl mice. Marked reduction in postprandial blood glucose concentration and obesity. Significant decrease in weight of mice intestines.	[137]
KK-A^y^ diabetic mice		Oral administration of black tea extracts for 5 weeks.	Black tea suppressed increased plasma glucose and insulin levels. Lowered plasma lipid level and white adipose tissue weight. It decreased the expression of tumor necrosis factor-α (TNF-α), interleukin-6 (IL-6), and monocyte chemotactic protein-1 (MCP-1) while increasing acetyl-CoA carboxylase (ACC) and carnitine palmitoyltransferase-1 (CPT-1).	[140]
High-fat diet-induced C57BL/6J male mice		Administration of black tea *ad libitum* for 14 weeks.	Black tea facilitated glucose uptake activity and promoted the translocation of glucose transporter 4 (GLUT 4) to the plasma membrane in muscle. Inhibited the high-fat diet-induced depletion of GLUT4, IRβ, and AMPKR levels.	[142]
Adults with T2D	Clinical trial	Daily ingestion of 200 or 600 mL of black tea for 12 weeks.	Black tea consumption significantly decreased glycated hemoglobin (HbA1c) levels. Elevated T cell-associated CD3+, CD4+, CD25+, FOXP3, and Th17 cell-associated CD3+ CD4+ IL-17+. Reduced Th1-associated CD3+ CD4+ IFN-Υ+ cells.	[147]
Orally sucrose-loaded normal and prediabetes male and female adults.		Random ingestion of 50 g of sucrose solution with 110 mg of black tea polymerized polyphenol (BTPP), 220 mg BTPP of black tea drink, or placebo.	Black tea ingestion significantly reduced postprandial blood glucose after sucrose ingestion.	[145]
Orally glucose-loaded healthy human adults		1 g of instant black tea and 75 g of glucose in 250 mL of water was ingested by subjects. 0.052 g of caffeine replaced tea for positive control.	Black tea drink significantly reduced plasma glucose concentrations at 120 min relative to the control and caffeine drinks. Increased insulin concentrations compared with the control and caffeine drinks at 90 min.	[144]
Healthy Japanese subjects		A one-off ingestion of 200 mL of black tea or placebo and 200 g of cooked rice.	Black tea drink significantly inhibited α-glucosidase, α-amylase, and sucrase enzymes. Significantly decreased the incremental area under the curve (AUC) of glucose and insulin.	[146]

### 6.5. Pu-erh Tea

The antidiabetic properties of pu-erh tea are summarized in Table 5.

#### 6.5.1. *In Vitro* Studies

Wang et al. investigated pu-erh tea’s phytochemical composition under the direction of biological activity [149]. From the pu-erh tea’s aqueous extract, one brand-new flavanol called (-)-epicatechin-3-O-(Z)-coumarate (**1**) and 16 well-known analogs (2–17) ((–)-epicatechin-3-*O*-(*E*)-coumarate (**2**)**,** (–)-epicatechin-3-*O*-(*E*)-caffeate (**3**)**,** (+)-catechin (**4**)**,** ampelopsin (**5**)**,** (–)-epicatechin (**6**)**,** (–)-epiafzelechin (**7**)**,** (–)-epicatechin-3-*O*-gallate (**8**)**,** (–)-epiafzelechin-3-*O*-gallate (**9**)**,** (+)-catechin-3-*O*-gallate (**10**)**,** (+)-epiafzelechin-3-*O*-gallate (**11**)**,** epicatechin- 3-*O*-*p*-hydroxybenzoate (**12**)**,** (–)-epigallo-catechin (**13**)**,** (±)-gallocatechin (**14**)**,** (–)-epigallo-catechin-3-*O*-gallate (**15**)**,** (+)-gallocatechin-3-*O*-gallate (**16**), and (–)-epicatechin-3-*O*-(3′′-*O*-methyl)-gallate (**17**)) were identified. By using spectroscopy and chemical analysis, their structures were discovered. The water extract of pu-erh tea and its fractions also demonstrated inhibitory effects toward α-glucosidases and lipases *in vitro*. Similarly, Du et al. [150] reported the inhibitory properties of the aqueous extract of pu-erh tea on the activities intestinal sucrase, maltase, and porcine pancreatic amylase. This was further supported by Huang et al. [151] and Gu et al. [152], who reported the ability of the ethyl acetate fraction (EF), 95% ethanol precipitate (EP), and puerin III (P3) in pu-erh tea to inhibit the activities of α-glucosidase *in vitro*.

#### 6.5.2. *In Vivo* Studies

Wu et al. investigated the effect of theabrownin from pu-erh tea and swinging exercise on overweight rats with insulin resistance [153]. Rats given a diet high in fat, sugar, and salt were orally administered theabrownin, rosiglitazone, or lovastatin (controls). Swinging and theabrownin significantly enhanced the rats’ blood lipid profiles and modulated insulin resistance and body weight. Theabrownin was further shown to improve the breakdown of nutrients and ingestion of lipids and carbohydrate via increased cyclic adenosine monophosphate (CAM) quantities and activation of insulin, protein kinase A, adenosine monophosphate-activated protein kinase, and circadian rhythm signaling pathways. Physical activity led to increased serum dopamine levels. This indicates a synergistic effect of exercise and theabrownin on improved metabolic syndrome and body weight.

The antidiabetic properties of theabrownin were further demonstrated in Goto–Kakizaki (GK) rats [154]. Yu et al. reported decreased body weight, FBG, and triglyceride levels in diabetic GK rats treated with theabrownin. Theabrownin also modulated adiponectin (ADPN) and leptin levels and glucokinase, hepatic lipase (HL), and hormone-sensitive triglyceride lipase (HSL) activities while improving intestinal microflora.

Intragastric administration of the water extract of pu-erh tea to db/db mice improved insulin response and glucose tolerance [150].

In another study, pu-erh tea was shown to cause a reduction in TG, cholesterol, insulin, and leptin levels while downregulating hepatic FAS mRNA and protein levels and enhancing AMPK phosphorylation in fructose-induced hyperlipidemia and hyperleptinemia [138]. The ability of pu-erh tea to upregulate AMPK phosphorylation, while phosphorylating Akt, leading to GLUT4 translocation, has also been demonstrated in skeletal muscles [129,155]. AMPK phosphorylation, elevated uncoupling protein 1 (UCP1), and insulin-like growth factor binding protein 1 (IGFBP-1) protein levels in WAT in pu-erh tea also suggest its potential to modulate metabolic syndrome and improve WAT browning [155].

Pu-erh tea has been reported to have a protective effect against diabetic nephropathy [155]. This has been attributed to its ability to reduce serum creatinine, urine albumin, and the mesangial matrix in db/db mice, with a concomitant reduction in glyoxalase I expression, thereby insinuating a reduction in AGE production in diabetic kidneys.

The antidiabetic properties of pu-erh tea improved with ripening. Ripened pu-erh tea (RIPT) displayed significant reductions in FBG and postprandial blood glucose levels in diabetic rats compared to unripened (raw) pu-erh tea [156]. RIPT also improved the intestinal microflora in diabetic rats.

#### 6.5.3. Clinical Trials

Daily consumption of pu-erh tea (333 mg three times per day in the form of a capsule) by individuals with metabolic syndrome led to reduced body weight and lower BMI [157]. Huang et al. [151] also demonstrated the ability of pu-erh tea to reduce hypercholesterolemia and hyperlipidemia in healthy individuals. Daily consumption of 300 mL of the tea infusion (50 mg/kg/day) for 4 weeks significantly improved gut metabolism, with concomitant modulation of cholesterol and lipid levels.

These clinical trials may give credence to the potential of pu-erh tea in the management of body weight and other metabolic activities linked to metabolic syndrome.

**Table 5:** Antidiabetic studies of pu-erh tea.

Cell Lines/Tissue/Animal Model/Human	Study	Treatment	Antidiabetic Mechanism	Reference
Enzyme inhibition	*In vitro*	Puerin III from pu-erh tea *α*-glucosidase.	By stifling the activity of the α-glucosidase enzyme.	[152]
		Different solvent fractions of pu-erh tea inhibitory effects.	Extract and fractions of pu-erh tea exhibited inhibitory activities against α-glucosidases and lipases.	[149]
		Inhibitory effects of (−)-epigallo-catechin-3-O-gallate on sucrose and maltase enzymes, a compound isolated from ethyl acetate fraction of a pu-erh tea water extract.	It showed moderate inhibitory effect against sucrase (IC_50_ = 32.5 μmol/L) and significant inhibitory effect against maltase (IC_50_ = 1.3 μmol/L).	
		α-glycosidase inhibitory property of different solvent fractions of pu-erh tea.	All the extracts showed concentration-dependent inhibition on α-glycosidase at 125 µg/mL.	[158]
		Water extract on HepG2 cells, intestinal sucrase, maltase, and porcine pancreatic amylase.	It decreased the activity of the enzymes rat intestine sucrase and maltase and pig pancreatic amylase while also substantially raising the absorption of glucose by HepG2 cells.	[150]
Male Wistar rats	*Ex vivo*	Pu-erh tea hot-water extract for 30 min on isolated rat epitrochlearis muscle of 5-week-old male Wistar rats.	In the absence of insulin, it increased the rate of 3-O-methylD-glucose (3MG) transport and the phosphorylation of Akt at Ser473, but it had no impact on the phosphorylation of AMPK.	[129]
Sprague–Dawley rats	*In vivo*	Administration of theabrownin (0.2812 g/kg BW, 0.5625 g/kg BW, and 1.125 g/kg BW) together with a swinging exercise routine of 30 min each day.	Swinging and theabrownin were found to drastically enhance rats’ blood lipid profiles and stop the development of insulin resistance and obesity.	[153]
Goto–Kakizaki (GK) rats		Intragastric administration of theabrownin.	In contrast to the significantly greater levels of circulating adiponectin (ADPN), leptin, and glucokinase in the serum, the subjects’ body weight, triglyceride (TG) content, fasting blood glucose (FBG), and HOMA-IR scores were all significantly lower. The number of distinct intestinal bacteria significantly increased, which is advantageous for the metabolism of lipids and glucose.	[154]
Wistar rats		Pu-erh tea was incorporated into the feed.	It reduced the hyperlipidemia and hyperleptinemia caused by fructose by decreasing FAS protein levels and increasing AMPK activation.	[138]
		Comparative study on ripened pu-erh tea (RIPT, with pile fermentation) and raw pu-erh tea (RAPT) in rats.	When comparing fasting blood glucose (FBG) and two-hour postprandial blood glucose (2h-PBG), ripened pu-erh tea extract outperformed raw pu-erh tea extract in terms of its antidiabetic properties. Ripe pu-erh tea encouraged the growth of several probiotics, including lactobacillus, the Prevotellaceae NK3B31 group, Alloprevotella, as well as Prevotella.	[156]
Mice		High-fat, high-sugar diet-induced ApoE-/- mice were given oral doses of puerin III, an isolated compound from Pu-erh tea, for 6 weeks.	It reduced the hypercholesterolemic and hyperglycemic ApoE-/- mice’s increased plasma total cholesterol and fasting blood glucose (FBG) levels.	[152]
		Administered as tea to male ICR mice for 7 days.	White adipose tissue AMP-activated protein kinase (AMPK) phosphorylation was increased, and visceral fat was substantially reduced without affecting body weight.	[159]
		Oral administration of pu-erh tea for 7 days, instead of drinking water, on excised skeletal muscle from male ICR mice.	It enhanced the translocation of GLUT4 to the skeletal muscle plasma membrane and phosphorylated AMP-activated protein kinase (AMPK), PI3K, and Akt/protein kinase B.	[155]
		Intragastric administration of water extract to db/db mice for 4 weeks.	It alleviated delayed insulin response and improved poor glucose tolerance.	[150]
		Administration of pu-erh tea to db/db mice for 8 weeks.	It inhibited the buildup of advanced glycation end products brought on by diabetes and reduced the expression of AGE receptor in glomeruli.	[160]
		Male ICR mice were given different solvent extracts of pu-erh tea orally.	In diabetic mice, postprandial hyperglycemia was significantly decreased by all extracts (at a dose of 50 mg/kg body weight).	[158]
Male patients	Clinical trial	For three months, metabolic syndrome patients (male) received either 333 mg/mL of aqueous tea extract or a placebo in the form of tea.	It resulted to a decrease in BMI, notable gains in body weight, and no appreciable changes in cholesterol, triglycerides, or HbA1c.	[157]
Healthy males and mice		Male humans and 3-week-old mice were given theabrownin via oral administration.	The intestinal FXR-FGF15 signaling pathway was inhibited by theabrownin, which raised ileal-conjugated bile acid levels. This led to a reduction in hepatic cholesterol and inhibited lipogenesis while also increasing bile acid synthesis and fecal excretion.	[151]

### 6.6. Summarized Antidiabetic Activities of White, Green, Oolong, Black, and Pu-erh Teas

**Figure 7:**
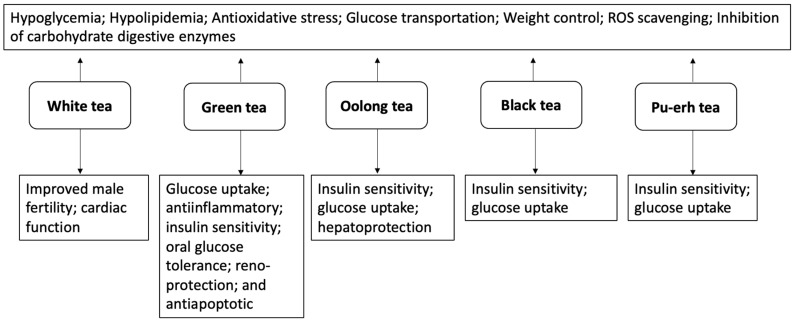
Summarized antidiabetic activities of white, green, oolong, black, and pu-erh teas.

A brief summary of the antidiabetic activities of white, green, oolong, black, and pu-erh teas is presented in Figure 7.

## 7. Potential Toxic Effects of Teas

Although teas have a relatively low toxicity risk, especially when consumed as a beverage, high doses or concentrated extracts have been linked to adverse effects, including hepatotoxicity [161], gastrointestinal problems [162], and potentially reduced efficacy of certain medications such as the beta-blockers, nadolol, and the cholesterol-lowering drug atorvastatin [163,164]. The phytochemical constituents of teas, such as EGCG, have been reported for their hepatoxic effect in mice (125 mg/kg, IP, single dose) [165]. Furthermore, the presence of caffeine in teas has been implicated in exacerbated effects of aminophylline, pseudoephedrine, ephedrine, and theophylline but suppresses the effect of adenosine [162].

## 8. Conclusions

Tea has been used for centuries as both a beverage and medicinal remedy. Research findings from various studies as highlighted in our review show the pharmacological potential of the major types of tea consumed in preventing and managing diabetes and its associated complications. These studies, which include *in vivo*, *in vitro*, *ex vivo*, and *in silico* analyses, as well as clinical trials, demonstrate the benefits of tea extracts, which contain active components such as phytochemicals, particularly polyphenols (flavanols), polysaccharides, and amino acids.

Generally, the studied teas exhibit hypoglycemic and hypolipidemic properties by suppressing blood glucose, cholesterol, triglyceride, and LDL levels. They are potent inhibitors of carbohydrate-digestive enzymes. Their antioxidant properties are characterized by their ability to scavenge free radicals and improve antioxidant enzyme activities while inhibiting lipid peroxidation.

Bioactive compounds including ellagitannins and catechins found in green tea are primarily responsible for its strong inhibitory effects on the activities of α-amylase and α-glucosidase. Extracts from teas exhibit antidiabetic properties by alleviating oxidative stress, managing hyperlipidemia, improving insulin sensitivity, and enhancing glucose tolerance. Additionally, they help mitigate diabetes-related consequences on the heart and brain, enhance myocardial acetate and alanine contents, and modulate the expression of glucose transporters (GLUT1 and GLUT3). White tea has the potential to improve reproductive dysfunctions associated with prediabetes by enhancing the antioxidant capabilities of the testes. Oolong tea may promote antidiabetic and anti-obesogenic activities by improving insulin sensitivity, GLUT4 translocation, lipid metabolism, and modulation of hepatic antioxidant enzyme activities.

EGCG found in teas may help prevent weight gain by improving glucose tolerance. Theaflavin, one of the main ingredients in black tea, improves glucose absorption and GLUT4 translocation, increasing insulin sensitivity. Theabrownin from pu-erh tea improves FBG, insulin sensitivity, lipid metabolism, and body weight.

However, detailed studies are needed in understanding the exact physiological mechanisms of actions of the various tea extracts in the pathways implicated in diabetes, as most of the studies reviewed did not detail the exact modes of action of these tea extracts. The bioactive principles in tea influence the pharmacological properties of its extract. Therefore, to clarify the key antidiabetic compounds present, it is essential to isolate and test purified individual bioactive compounds found in these teas.

In conclusion, easily available teas can be used as an affordable source of antidiabetic treatments, presenting a promising opportunity for cost reduction. These beneficial remedies will be more accessible to individuals from middle- and low-income backgrounds and enable effective health management. By including teas into their daily routines like they would other beverages, patients can find a practical solution that supports their well-being without the financial burden often associated with conventional pharmaceutical drugs.
